# Taxonomy of the ant genus *Cardiocondyla* Emery, 1869 from China, with the description of a new species of the *sima* group (Hymenoptera, Formicidae)

**DOI:** 10.3897/zookeys.1276.183523

**Published:** 2026-04-03

**Authors:** Yi Huang, Ying Zhong, Tianqi Wu, Yuyuan Huang, Li Tian

**Affiliations:** 1 Department of Entomology and MOA Key Lab of Pest Monitoring and Green Management, College of Plant Protection, China Agricultural University, Beijing, China Fujian Agriculture and Forestry University Fuzhou China; 2 GCTB-NSU Joint Institute of Technology, Guangzhou College of Technology and Business, Foshan, Guangdong, China College of Plant Protection, China Agricultural University Beijing China; 3 Shenzhen School of The Affiliated High School of Peking University, Shenzhen, Guangdong, China GCTB-NSU Joint Institute of Technology, Guangzhou College of Technology and Business Foshan China; 4 Fujian Agriculture and Forestry University, Fuzhou, Fujian, China Shenzhen School of The Affiliated High School of Peking University Shenzhen China

**Keywords:** Chinese fauna, key, Scanning Electron Microscope, worker morphology

## Abstract

The genus *Cardiocondyla* Emery, 1869 is a diverse group of ants in the subfamily Myrmicinae, with a total of 89 recognised species and subspecies recorded worldwide. A new species of the *sima* group *C. zhoui***sp. nov**. is described herein, from China, based on worker morphology. The main diagnostic characters of this new species are as follows: 11-segmented antennae; occiput partly smooth and shining; clypeus broad and robust; mesosomal dorsum strongly convex and mostly smooth; metanotal groove absent; and petiolar peduncle long and slender. The new species is similar to *C. sima* Wheeler, 1935, but it differs in a smooth and shining occipital surface, evenly convex anterior clypeal margin, more elevated mesosomal outline, and more elongated petiolar peduncle. Additionally, a new synonymy is proposed: *C. insutura* Zhou, 2001, **syn. nov**. (= *C. minutior* Forel, 1899). Scanning Electron Microscope (SEM) images and high-resolution colour images of Chinese *Cardiocondyla* species are provided, with a taxonomic key to the Chinese *Cardiocondyla* species based on worker caste.

## Introduction

The genus *Cardiocondyla* Emery, 1869 currently contains 87 valid species and two valid subspecies (Bolton 2026). *Cardiocondyla* ants typically have small colonies and nest entrances that are small and concealed by substrate, making nests difficult to detect (Seifert 2003). Species within the genus are native to the Old World, but at least five species have been introduced to the New World through human activity (Wetterer 2014). In addition to the winged males, several species of this genus have been observed to possess an “ergatoid” male caste characterised by small eyes and underdeveloped ocelli (Kugler 1984; Seifert 2003; Heinze et al. 2005). Studies suggest that this male polymorphism is a plesiomorphic trait, and the loss of winged males has occurred convergently in multiple lineages (Heinze et al. 2005).

**Figure 1.**
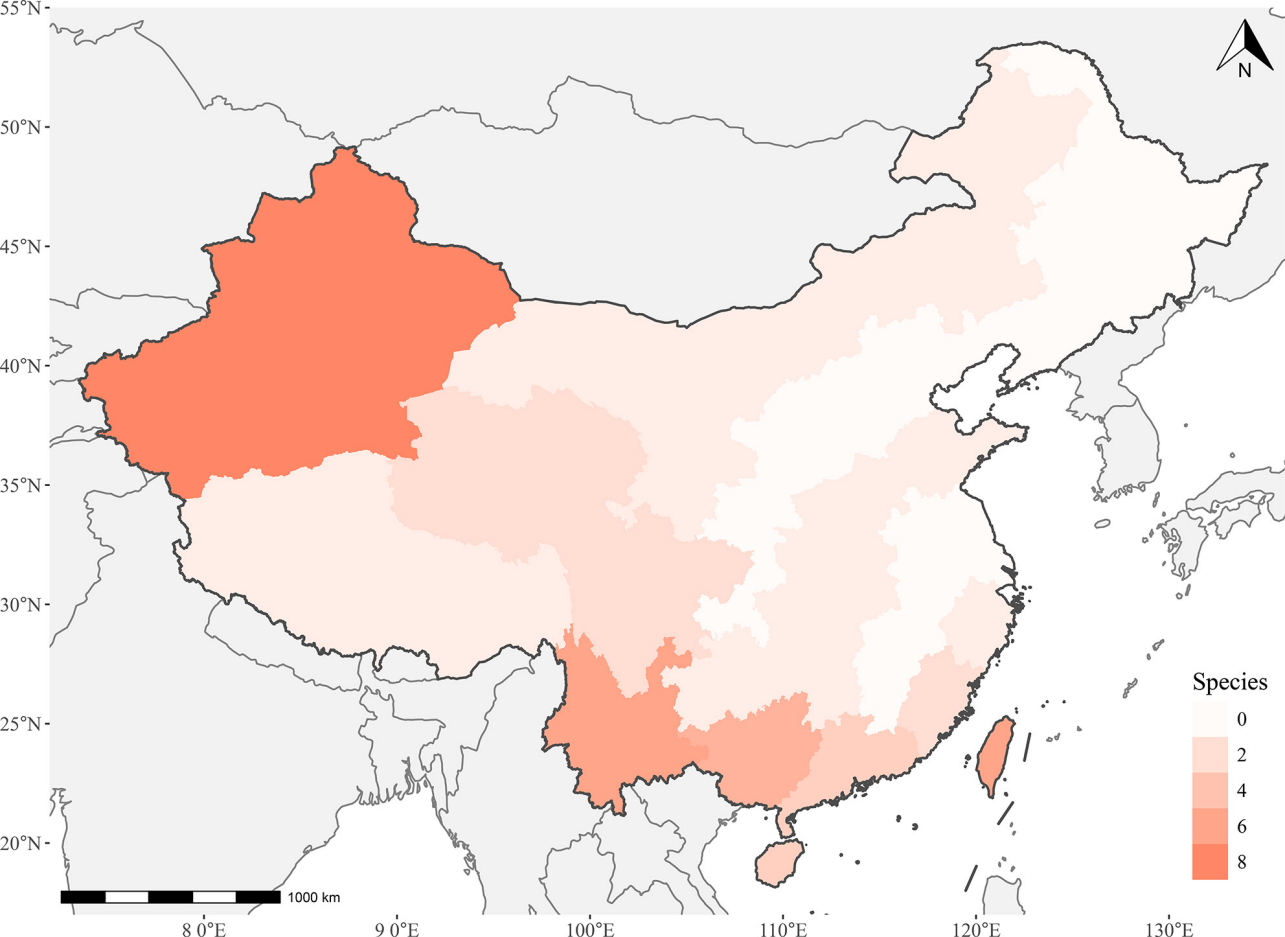
Distribution map of *Cardiocondyla* species in China. Coloured areas represent provincial-level administrative divisions. White areas indicate provinces with no recorded distribution of the genus. Red areas indicate provinces where species are present, with deeper shades corresponding to a higher number of species.

Taxonomically, a preliminary classification of the genus *Cardiocondyla* in China and a taxonomic key to two species were provided by Wu and Wang (1995). In his classification study of ant species in Guangxi Province, Zhou (2001) described a new species, *C. insutura* Zhou, 2001, syn. nov. (= *C. minutior* Forel, 1899). Subsequent study of ants from Taiwan Province revealed the distribution of four species: *C. obscurior* Wheeler, 1929, *C. wroughtonii* Forel, 1890, *C. minutior* Forel, 1899, and *C. kagutsuchi* Terayama, 1999, for which Terayama (2009) provided a key to distinguish them. Of these, *C. kagutsuchi* was described by Terayama (1999) in his taxonomic study of Japanese *Cardiocondyla*. That study also described *C. tsukuyomi* Terayama, 1999 (regarded as a junior synonym of *C. minutior* by Seifert (2023b)) and *C. yamauchii* Terayama, 1999 (regarded as a junior synonym of *C. wroughtonii* (Forel, 1890) by Seifert (2024), although they were diagnosed as having colour differences). Seifert (2003) noted that the taxonomic knowledge of *Cardiocondyla* in the Oriental, Indo-Malayan, and Australasian faunal regions remains significantly lacking. The taxonomic studies by Seifert (2023a) for the Oriental and Australasian faunal regions have been partially organised and clarified, but the taxonomy of this genus in the Indo-Malayan region remains to be thoroughly investigated. Additional taxonomic studies have focused on male morphology in *Cardiocondyla* (Kugler 1984; Ramamonjisoa et al. 2024). To date, a total of 16 species of *Cardiocondyla* have been recorded in the literature within China, exhibiting high diversity in the northwestern and southwestern regions of the country (Fig. 1).

The *sima* group within the genus *Cardiocondyla* was originally classified as an independent genus *Prosopidris* (Reiskind 1965). Currently, the genus *Prosopidris* has become a junior synonym of the genus *Cardiocondyla* (Meggers et al. 1973; Krombein et al. 1979). The *sima* group includes two species: *C. sima* Wheeler, 1935 and *C. papuana* (Reiskind, 1965). The species group is easily identified by the 11-segmented antennae and larger antennal clubs compared to the other 12-segmented antennae species of the genus (Reiskind 1965).

A new species of the genus *Cardiocondyla* from Yunnan Province, China, is described herein, which is the first record of the *sima* group in China. This species can be easily distinguished from other species of the genus in China by its different number of antennal segments and the elevated promesonotum. The species can be allocated to the *sima* group due to its 11-segmented antennae and the larger antennal clubs compared to other *Cardiocondyla* species (Reiskind 1965).

## Materials and methods

All type and non-type material examined in this study is deposited in the Entomological Museum of China Agricultural University (**CAU**), Beijing, China. Specimens were mounted on white triangular pieces and labelled with a red label for the holotype and yellow labels for the paratypes.

Examination and identification of type specimens were carried out using a Phenix XSP-02 microscope, multifocal images were taken using a Samsung SM-N9860 telephoto camera. Photographs were processed and stacked using the software Adobe Photoshop and Helicon Focus. Examination and identification of non-type specimens were carried out using a Nikon SMZ18 microscope, multifocal images were taken using a Canon EOS 7D camera, and photographs were processed and stacked using the software Adobe Photoshop and Helicon Focus. Measurements of type specimens were taken using the software HAYEAR coupled with a HY-800B digital camera attached to a 0745 lens. All morphological line drawings were prepared using Adobe Illustrator. All scanning electron microscope (SEM) photographs were acquired using a HITACHI SU8010 SEM at 5kV (Institute of Food Science and Technology, Chinese Academy of Agricultural Science, Beijing, China). Standardised morphological, sculptural and pilosity terminology follows Bolton (1994), Harris (1979), and Wilson (1955), respectively.

Standard measurements and indices for the new species follow the definitions of Bolton (1982). Refinements to the list of definitions are adapted from Sharaf et al. (2024), and supplemented with the measurements FRW, PSL, PI, PHI, PPI, PPHI, and WI. All measurements are expressed in millimetres (mm):

**EL** Eye length: maximum diameter of the compound eye in lateral view.

**FRW** Frontal lobes width: maximum distance between the outermost margins of the frontal lobes in full-face view.

**HL** Head length: maximum length of the head in full-face view, measured from the midpoint of the anterior clypeal margin to the midpoint of the occipital margin, excluding the mandibles.

**HW** Head width: maximum width of the head in full-face view, excluding the eyes.

**ML** Mesosoma length: diagonal length of the mesosoma in lateral view, measured from the point between the cervical shield and pronotum to the posteriormost margin of the propodeal lobe.

**PH** Petiole height: maximum height of the petiole in lateral view, measured perpendicularly from the dorsal summit to the ventral margin.

**PL** Petiole length: maximum length of the petiole in lateral view, measured from the anteroventral corner to the posteriormost margin.

**PPH** Postpetiole height: maximum height of the postpetiole in lateral view.

**PPL** Postpetiole length: maximum length of the postpetiole in lateral view.

**PPW** Postpetiole width: maximum width of the postpetiole in dorsal view.

**PRW** Pronotal width: maximum width of the pronotum in dorsal view.

**PSL** Propodeal spine length: vertical length of the propodeal spine in lateral view, measured perpendicularly from its basal constriction to its apex.

**PW** Petiole width: maximum width measured in dorsal view.

**SL** Scape length: maximum straight-line length of the antennal scape, excluding the basal condyle.

**CI** Cephalic index: HW × 100 / HL.

**SI** Scape index: SL × 100 / HW.

**PI** Petiolar index: PW × 100 / PL.

**PHI** Petiolar height index: PW × 100 / PH.

**PPI** Postpetiolar index: PPW × 100 / PPL.

**PPHI** Postpetiolar height index: PPW × 100 / PPH.

**WI** Waist index: PPW × 100 / PW.

The following abbreviations are used to indicate collection institutions:

**ABS** Archbold Biological Station, Lake Placid, FL, U.S.A.

**AMNH** American Museum of Natural History, Central Park West at 79^th^ Street, New York, U.S.A.

**CASC** California Academy of Sciences, San Francisco, California, U.S.A.

**CAU** Entomological Museum of China Agricultural University, Beijing, China.

**CDRS** Charles Darwin Research Station, Puerto Ayora on Santa Cruz Island, Galápagos Islands, Ecuador.

**DBBC** Douglas B. Booher Collection.

**EcoFoG** Ecology of Guiana Forests, UMR (Joint Research Unit) AgroParisTech, Cirad, CNRS, INRAE, Université des Antilles et Université de la Guyane.

**EMEC** Essig Museum of Entomology, University of California, Berkeley, U.S.A.

**EMSC** Eli M. Sarnat Collection.

**FMNH** Field Museum of Natural History, Chicago, U.S.A.

**GNUC** Guangxi Normal University, Guilin, Guangxi, China.

**KSMA** King Saud Museum of Arthropods, King Saud University, Riyadh, Kingdom of Saudi Arabia.

**LACM** Los Angeles County Museum of Natural History, 900 Exposition Boulevard, Los Angeles, California, U.S.A.

**MSNG** Museo Civico di Storia Naturale “Giacomo Doria.”, Genoa, Italy.

**NHMB** Naturhistorisches Museum, Augustinergasse, Basel, Switzerland.

**NHMW** Naturhistorisches Museum Wien, Postfach, Burgring, Vienna, Austria.

**NMHUK** Natural History Museum, London, United Kingdom.

**PSWC** Philip S. Ward Collection, University of California, Davis, California, U.S.A.

**SMNG** Senckenberg Museum für Naturkunde Görlitz, Görlitz, Germany.

**UCDC** University of California, Davis. R.M. Bohart Museum of Entomology, Davis, California, U.S.A.

**USMN** United States National Museum of Natural History, Washington D.C., U.S.A.

**ZMGU** Zoological Museum of Gorgan University, Gor-gan, Iran.

## Systematics

### Genus Cardiocondyla

Taxon classification

Animalia

HymenopteraFormicidae

Emery, 1869

6EC4C8BD-2FF3-5157-A2B0-9D2970F7DAF6

Cardiocondyla Emery, 1869.
= Dyclona Santschi, 1930.
= Emeryia Forel, 1890.
= Loncyda Santschi, 1930.
= Prosopidris Wheeler, 1935.
= Xenometra Emery, 1917.

### Synonymic list of Chinese Cardiocondyla species

*C. elegans* Emery, 1869.

= *C. santschii* Forel, 1905.

= *C. gallica* (Bernard, 1957).

= *C. provincialis* Bernard, 1956.

*C. gibbosa* Kuznetsov-Ugamsky, 1927.

*C. itsukii* Seifert et al., 2017.

*C. kagutsuchi* Terayama, 1999.

*C. koshewnikovi* Ruzsky, 1902.

*C. minutior* Forel, 1899.

= *C. breviscapus* Seifert, 2003.

= *C. insutura* Zhou, 2001, syn. nov.

= *C. tsukuyomi* Terayama, 1999.

*C. parvinoda* Forel, 1902.

*C. nigra* Forel, 1905.

= *C. bicoronata* Seifert, 2003.

= *C. torretassoi* Finzi, 1936.

*C. nuda* (Mayr, 1866).

*C. obscurior* Wheeler, 1929.

= *C. bicolor* Donisthorpe, 1930.

*C. rolandi* Seifert, 2023.

*C. stambuloffii* Forel, 1892.

= *C. bogdanovi* Ruzsky, 1905.

= *C. montandoni* Santschi, 1912.

= *C. stambuloffii taurica* Karavaiev, 1927.

*C. tibetana* Seifert, 2003.

*C. uljanini* Emery, 1889.

= *C. elegans schkaffi* Alpatov & Arnol’di, 1928.

*C. wroughtonii* (Forel, 1890).

= *C. emeryi chlorotica* Menozzi, 1930.

= *C. longispina* Karavaiev, 1935.

= *C. wroughtonii bimaculata* Wheeler, 1929.

= *C. wroughtonii hawaiensis* Forel, 1899.

= *C. wroughtonii quadraticeps* Forel, 1912.

= *C. yamauchii* Terayama, 1999.

*C. zhoui* sp. nov.

### The *batesii* group

### Cardiocondyla nigra

Taxon classification

Animalia

HymenopteraFormicidae

Forel, 1905

EF594B28-D831-59E0-BF4F-2BB8B263C356

Cardiocondyla batesii var. nigra Forel, 1905: 174 (worker and queen), Tunisia [images examined]. Male and ergatoid male described: Santschi 1907: 324. Initially raised to species: Schembri and Collingwood 1981: 428. Synonymised with C. batesii Forel, 1894: Radchenko 1995: 451. Re-raised to species: Schembri and Collingwood 1995: 154.
Cardiocondyla elegans var. torretassoi Finzi, 1936: 167 (worker), Egypt [images examined]. Synonymised: Seifert 2003: 240.
Cardiocondyla bicoronata Seifert, 2003: 242, figs 21, 22 (worker and queen), Jordan [images examined]. Synonymised: Seifert 2023b: 46.

#### Type material examined.

**Tunisia** • 1 syntype worker, Kairouan, 35°40'41"N, 10°05'47"E, F. Santschi leg. (MHNG, CASENT0908336) [images examined].

#### Non-type material examined.

**Cape verde** • 1 worker, São Vicente, foot of Verde Mt., 200 m elev., 17-xii-1978, Winter leg. (BMNH, CASENT0281805) [images examined]. **Cyprus** • 1 worker, 34°44'40"N, 32°58'33"E, 01-iii-2003, A. Schrempf leg. (SMNG, ANTWEB1048524) [images examined].

#### Distribution.

Xinjiang (Xia and Zheng 1997), Qinghai (Tie and Xu 2004).

#### Remarks.

The species *Cardiocondyla nigra* is the only known species of the *batesii* group in China, recorded from Xinjiang (Xia and Zheng 1997) and Qinghai (Tie and Xu 2004). However, most of the material we examined, including that examined by Seifert (2023b), is from around the Mediterranean region. Therefore, the records of this species in China deserve to be re-examined. The species can be recognised by the smooth, shining body and the clearly depressed metanotal groove. Among Chinese species it is most similar to *C. ulianini* Emery, 1889, but can be identified by the clear fine reticulation of the pronotum and the sculpture of the propodeum.

##### The *elegans* group

### Cardiocondyla elegans

Taxon classification

Animalia

HymenopteraFormicidae

Emery, 1869

D32794B5-E22D-5B84-9BE3-2965BB6C4D5E

Cardiocondyla elegans Emery, 1869: 21, pl. 1, fig. 10 (worker and queen), Italy [images examined]. Ergatoid male described: Menozzi 1918: 83; Kugler 1984: 14.
Cardiocondyla elegans r. santschii Forel, 1905: 174 (worker), France [images examined]. Initially synonymised: Bondroit 1918: 147. Returned to subspecies of C. elegans: Santschi 1926: 293. Re-synonymised: Bolton 1995: 133.
Cardiocondyla provincialis Bernard, 1956: 303, fig. 6 (worker), France [images examined]. Synonymised: Radchenko 1995: 449.
Xenometra gallica Bernard, 1957: 101, fig. 1 (ergatoid male), France [images examined]. Synonymised: Baroni Urbani 1973: 200.

#### Type material examined.

**Italy** • 1 lectotype worker, Naples, Bosco di Capodimonte, 40°52'00"N, 14°15'E, 18-vi-1866, C. Emery leg. (MSNG, CASENT0904460) [images examined].

#### Non-type material examined.

**Bulgaria** • 1 worker, Chernomorec, 42°27'N, 27°39'E, 22-vii-2006 to 02-viii-2006, L. Borowiec leg. (CASC, CASENT0179878) [images examined]. **France** • 1 worker, Brehemont-1.1 km E, Bank of Loire, 47°17'37"N, 00°20'29"E, 35 m elev., 15-vii-2010, J.C. Lenoir leg. (SMNG, ANTWEB1048511) [images examined]. **Italy** • 1 worker, Lombardy, Godiasco, 44°54'N, 09°03'E, 25-vi-1987, R. Sciaky leg. (PSWC) [images examined].

#### Distribution.

Xinjiang (Collingwood and Heatwole 2000).

#### Remarks.

The species *Cardiocondyla elegans* has been recorded in China only from the north-west, in Xinjiang (Collingwood and Heatwole 2000). Originally, the ergatoid male of this species was erroneously regarded as a new genus, *Xenometra*, by Bernard (1957), but Baroni Urbani (1973) later considered it to be a junior synonym. The junior synonym, *C. elegans santschii*, which is a subspecies of *C. elegans*, was described by Forel (1905) with reference to differences in body size, pronotal shoulders, eye position and second node. However, these examination of syntypes CASENT0917796 and CASENT0908353 shows that these characters are not consistently represented. These specimens exhibit a pair of upwardly directed propodeal spines (unobservable in CASENT0917796) and a more distinctive sculpture of the mesosoma. Following the synonymisation of *C. elegans santschii* by Bondroit (1918), Santschi (1926) further proposed distinct differences in the petiole and postpetiole, as well as thicker propodeal spines (except CASENT0917796). However, such differences were recorded by Emery (1909), and from the illustrations, it appears that these differences in the petiole and postpetiole may be due to different views. Therefore, these minor differences can be considered to be the result of some observational errors and variability in different geographical areas, and Bolton (1995) also continued to refer to this subspecies as a junior synonym of *C. elegans*.

##### The *minutior* group

### Cardiocondyla minutior

Taxon classification

Animalia

HymenopteraFormicidae

Forel, 1899

3E8FADE0-CDFD-55E3-B8BD-84A90DE5F8A3

Fig. 2

Cardiocondyla nuda var. *minutior* Forel, 1899: 120 (worker), Hawaii [images examined]. Queen described: Wheeler 1922: 317. Synonymised with C. nuda: Wilson and Taylor, 1967: 55. Raised to species: Seifert 2003: 283.
Cardiocondyla tsukuyomi Terayama, 1999: 101, figs 11–13 (worker, queen, male and ergatoid male), Japan [images examined]. Synonymised: Seifert 2003: 283.
Cardiocondyla insutura Zhou, 2001: 85, 233, figs 137, 138 (worker), China [holotype examined]. syn. nov.
Cardiocondyla breviscapus Seifert, 2003: 288, fig. 64 (worker), India [images examined]. Synonymised: Seifert 2023a: 26.

#### Type material examined.

**Hawaii** • 1 syntype worker, Oahu Is., Honolulu, 21°00'54"N, 156°45'57"W, 1893, R.C.L. Perkins leg. (BMNH, CASENT0102304) [images examined]. • 1 syntype worker, Molokai Mt., 914 m elev., 01-ix-1893, R.C.L. Perkins leg. (MHNG, CASENT0908344) [images examined].

**Figure 2.**
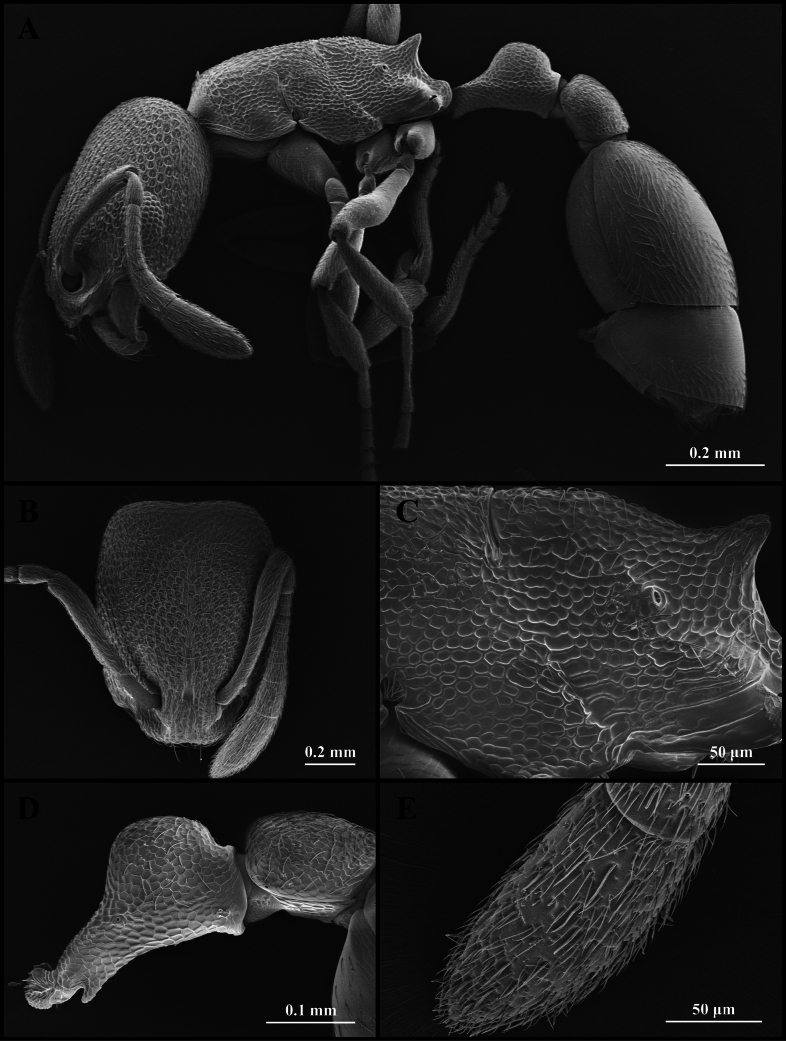
Scanning electron micrographs of *Cardiocondyla minutior* (worker) (CAU, CAU-ANT-2510-03, imaged destructively). **A**. Body, lateral view; **B**. Head, full-face view; **C**. Propodeum, lateral view; **D**. Petiole, lateral view; **E**. Apical antennomere.

#### Non-type material examined.

**Bahrain** • 1 worker, Bahrain Fort, 16-xii-2022, C. Georgiadis leg. (KSMA, ANTWEB1041912) [images examined]. **Brazil** • 1 worker, Amapá, Porto Grande, 00°34'19"N, 51°13'16"W, 64 m elev., 26-ii-2018, H. De Aguiar et al. leg. (EcoFoG, ECOFOG-OI17-0006-06) [images examined]. **California** • 1 worker, San Diego, 32°42'55"N, 117°09'26"W, 150 m elev., 18-ix-1982, M.S. Trepanier leg. (UCDC, CASENT0005679) [images examined]. **Costa Rica** • 1 worker, Puntarenas, 2 km E Tivives, 09°52'N, 84°42'W, 5 m elev., 06-vi-1992, L.S. Farley leg. (PSWC, CASENT0914963) [images examined]. **Ecuador** • 1 worker, Galapagos Iss, Isabela, Sierra Negra, 00°50'52"S, 91°09'48"W, 01-xii-1986, S. Abedrabbo leg. (CDRS, CASENT0173261) [images examined]. **Fiji** • 1 worker, Yasawa Prov., McDonald’s Resort, 17°20'56"S, 177°08'19"E, 55 m elev., 12-i-2005, E.M. Sarnat leg. (EMSC, CASENT0171077) [images examined]. **Florida** • 1 worker, Dade, Shark Valley, 25°40'N, 80°46'W, 05-xi-1989, Mark Deyrup leg. (ABS, CASENT0103740) [images examined]. **Hawaii** • 1 worker, Hilo, 19°43'21"N, 155°05'13"W, 08-ix-1958, J. Yoshiyama leg. (LACM, CASENT0103436) [images examined]. **Malaysia** • 1 worker, Melina Beach-Paya, Strand, 02°46'37"N, 104°07'05"E, 03-vi-2007 (SMNG, ANTWEB1048523) [images examined]. **Oman** • 1 worker, 23°32'37"N, 58°55'60"E, 280 m elev., 06-iv-2016, A. Polaszek leg. (KSMA, CASENT0922293) [images examined]. • 1 worker, Dhofar, Ayn Razat, 17°07'47"N, 54°14'11"E, 121 m elev., 20-xi-2017, M.R. Sharaf leg. (KSMA, CASENT0922873) [images examined]. **Palau** • 1 worker, Peleliu Is., Peleliu State, Main dock area, 07°02'55"N, 134°16'12"E, 05-viii-2008, J. E. Czekanski-Moir leg. (FMNH, FMNHINS0000065820) [images examined]. **Seychelles** • 1 worker, Cosmoledo Atoll, 09°45'11"S, 47°38'56"E, 10 m elev., 16-xi-2007 to 19-xi-2007, G. Galman leg. (CASC, CASENT0196173) [images examined]. • 1 worker, Cosmoledo Atoll, 09°44'07"S, 47°38'52"E, 10 m elev., 21-xi-2008 to 24-xi-2008, G. Galman leg. (CASC, CASENT0196227) [images examined]. **Yemen** • 1 worker, Socotra, Dixam, 12°29'36"N, 53°59'29"E, 656 m elev., 24-iv-2014, M.R. Sharaf leg. (CASC, CASENT0746617) [images examined]. **China** • 1 worker, Guangdong Province, Xuanhao Cheng leg. (CAU, CAU-ANT-2510-01); • 1 worker, Guangdong Province, Zhaoqing City, Xuanhao Cheng leg. (CAU, CAU-ANT-2510-02).

#### Worker description.

Head longer than wide, with weakly concave occipital margin and rounded lateral corners (Fig. 2B). Antennae short; scapes surpassing the midlength of the head but not reaching the occipital margin (Fig. 2B). Frontal lobes narrow (Fig. 2B). Anterior clypeal margin emarginate (Fig. 2B). Masticatory margin of mandible with five teeth. Dorsal outline of mesosoma flat (Fig. 2A). Metanotal groove absent (Fig. 2A, C). Mesopropodeal suture impressed as a narrow line (Fig. 2C). Propodeal spines short (Fig. 2C). Metapleural gland orifice slit-like (Fig. 2A). Petiolar peduncle short and stout. Petiolar node short (Fig. 2D). Postpetiole compressed, with ventral process weakly developed (Fig. 2D).

***Sculpture***. Frontal area centrally striate, surrounded by foveate-striate sculpture, the foveae shallow and often confluent (Fig. 2B). Frontal lobes and clypeus striate (Fig. 2B). Antennae smooth (Fig. 2B). Mesosoma alveolate (Fig. 2A, C). Petiole and postpetiole alveolate (Fig. 2D). Abdominal segments IV–VII smooth (Fig. 2A).

***Pilosity***. Body densely covered with short, appressed pubescence (Fig. 2A). Anterior clypeal margin with long, appressed hairs and three long, erect setae (Fig. 2B). Antennae bearing fine elongate hairs, and long flattened setae (Fig. 2E). Abdominal segments IV–VII with dense, long, appressed pubescence (Fig. 2A).

#### Distribution.

Hainan (Lee et al. 2020), Macau (Leong et al. 2017), Hong Kong (Guénard 2018), Taiwan (Terayama 2009), Guangxi (Zhou 2001), Guangdong (new record).

#### Remarks.

The species *Cardiocondyla minutior* was initially regarded as a subspecies of *C. nuda* by Forel (1903), later treated as a junior synonym of *C. nuda* by Wilson and Taylor (1967), before being elevated to species rank by Heinze (1999) and subsequently redescribed by Seifert (2003). *Cardiocondyla minutior* is recognised as a tramp ant species in China, with documented occurrences in Hainan (Lee et al. 2020), Macau (Leong et al. 2017), Hong Kong (Guénard 2018), and Taiwan (Terayama 2009). This species is also recorded for the first time from Guangdong, representing a new provincial record. Given the formidable human-mediated dispersal capacity characteristic of this genus, *C. minutior* may have expanded its distribution to additional provinces in southern China. The worker of *C. minutior* can be distinguished from that of *C. emeryi* by the following combination of characters: a much lower postpetiole (Fig. 2D), which lacks an anteroventral bulge; a much broader frontal carinae; a metanotal groove that is nearly absent (Fig. 2C); Abdominal tergite IV with significantly denser pubescence; longer pubescence on the abdominal tergites; and more developed microsetae on the eyes, the longest of which measure 6-10 μm (Seifert 2003). The species *C. insutura* syn. nov. is proposed here as a junior synonym of *C. minutior*. Upon examining the holotype of *C. insutura* syn. nov. (deposited in GNUC, holotype examined) and comparing it with the type material of *C. minutior* as well as a broad series of non-type specimens from its global range. The holotype of this new synonym shows no consistent morphological differences from *C. minutior*. Zhou (2001) originally distinguished *C. insutura* syn. nov. from *C. nuda* by the absence of a metanotal groove and the presence of propodeal spines. However, these characters are diagnostic for *C. minutior* as well.

### Cardiocondyla parvinoda

Taxon classification

Animalia

HymenopteraFormicidae

Forel, 1902

0078270E-01C0-5447-B682-B3CB95EF7132

Cardiocondyla parvinoda Forel, 1902: 213 (worker) India [images examined].

#### Type material examined.

**India** • 1 syntype worker, Poona, 18°30'48"N, 73°51'00"E, Wroughton leg. (MHNG, CASENT0908346) [images examined].

#### Non-type material examined.

**India** • 1 worker, West Bengal, Calcutta, 22°33'46"N, 88°21'47"E, 01-iii-1900, de Niceville and Bingham leg. (BMNH, CASENT0281807) [images examined]. **Thailand** • 1 worker, Mae Hong Son, vor Doi Chiang Dao, 19°11'24"N, 98°34'12"E, 16-i-2004 (SMNG, ANTWEB1048530) [images examined].

#### Distribution.

Taiwan (Seifert 2023a).

#### Remarks.

The species *Cardiocondyla parvinoda* was described by Forel in 1902 and was redescribed by Seifert (2023a). This species has documented distribution records only from Taiwan, China. This species has the largest size within the *minutior* group and can be distinguished from other known species by its pronotum, which in dorsal view is extensively smooth and shining. However, in specimen CASENT0281807, the characteristic extensively smooth and shining dorsum is absent, with the dorsum instead exhibiting coarse reticulate sculpture. Seifert (2023a) examined this specimen by photo evaluation and determined it to belong to *C. parvinoda*. The taxonomic status of specimen CASENT0281807 remains to be assessed.

##### The *nuda* group

### Cardiocondyla itsukii

Taxon classification

Animalia

HymenopteraFormicidae

Seifert et al., 2017

3A4BEB6E-E6D6-5519-9242-B35687393095

Figs 3, 4

Cardiocondyla itsukii Seifert et al., 2017: 339, figs 10–12 (worker, queen and male), Japan [images examined].

#### Type material examined.

**Japan** • Holotype and 1 paratype workers, Shizuoka, Iwata-shi, 34°43'23"N, 137°50'20"E, 12 m elev., 05-ix-2010, I. Okita leg. (SMNG, ANTWEB1038017; CASC, CASENT0922311) [images examined].

**Figure 3.**
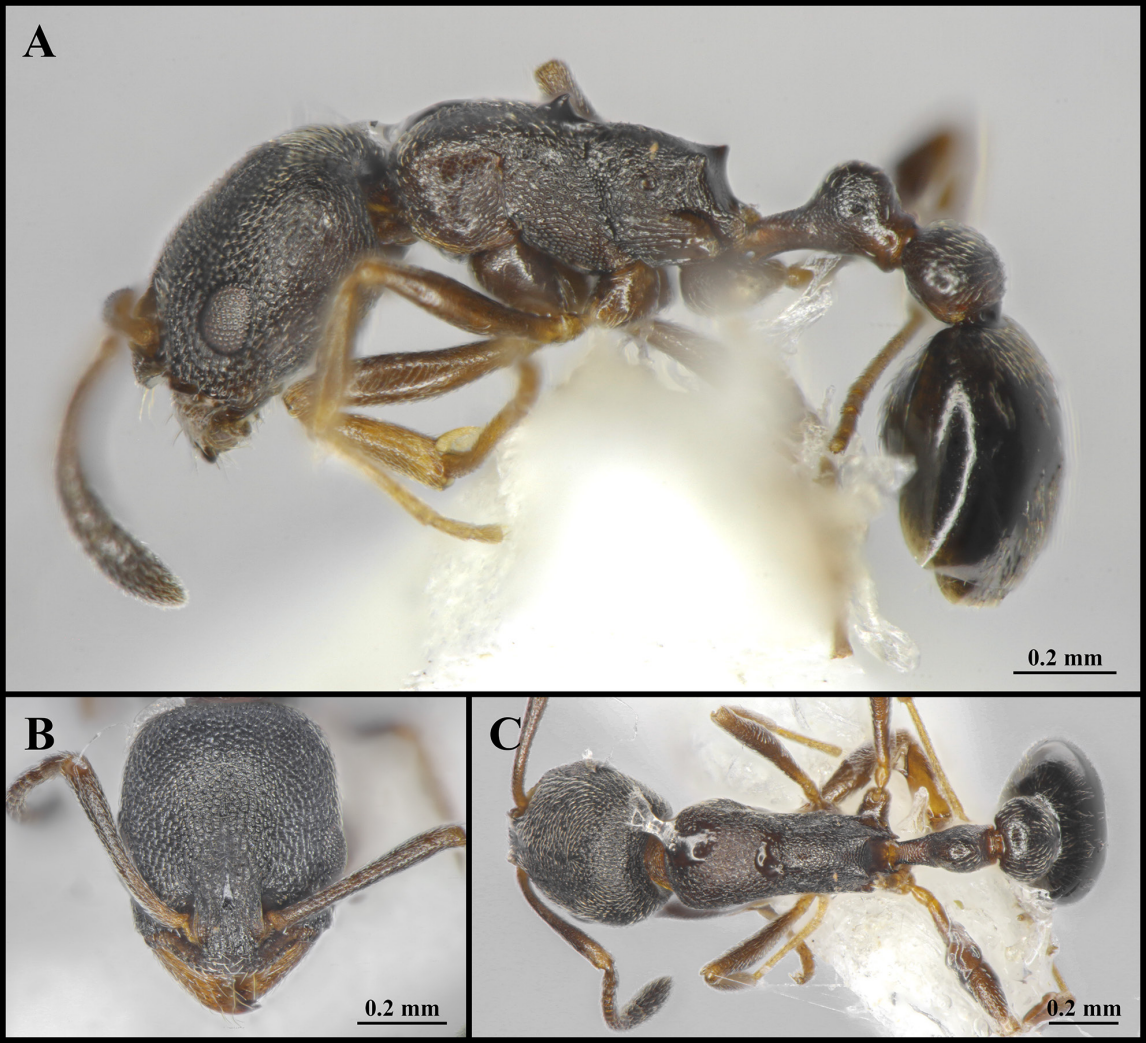
Worker of *Cardiocondyla itsukii* (CAU, CAU-ANT-2520-01). **A**. Body in lateral view; **B**. Head in full-face view; **C**. Body in dorsal view.

**Figure 4.**
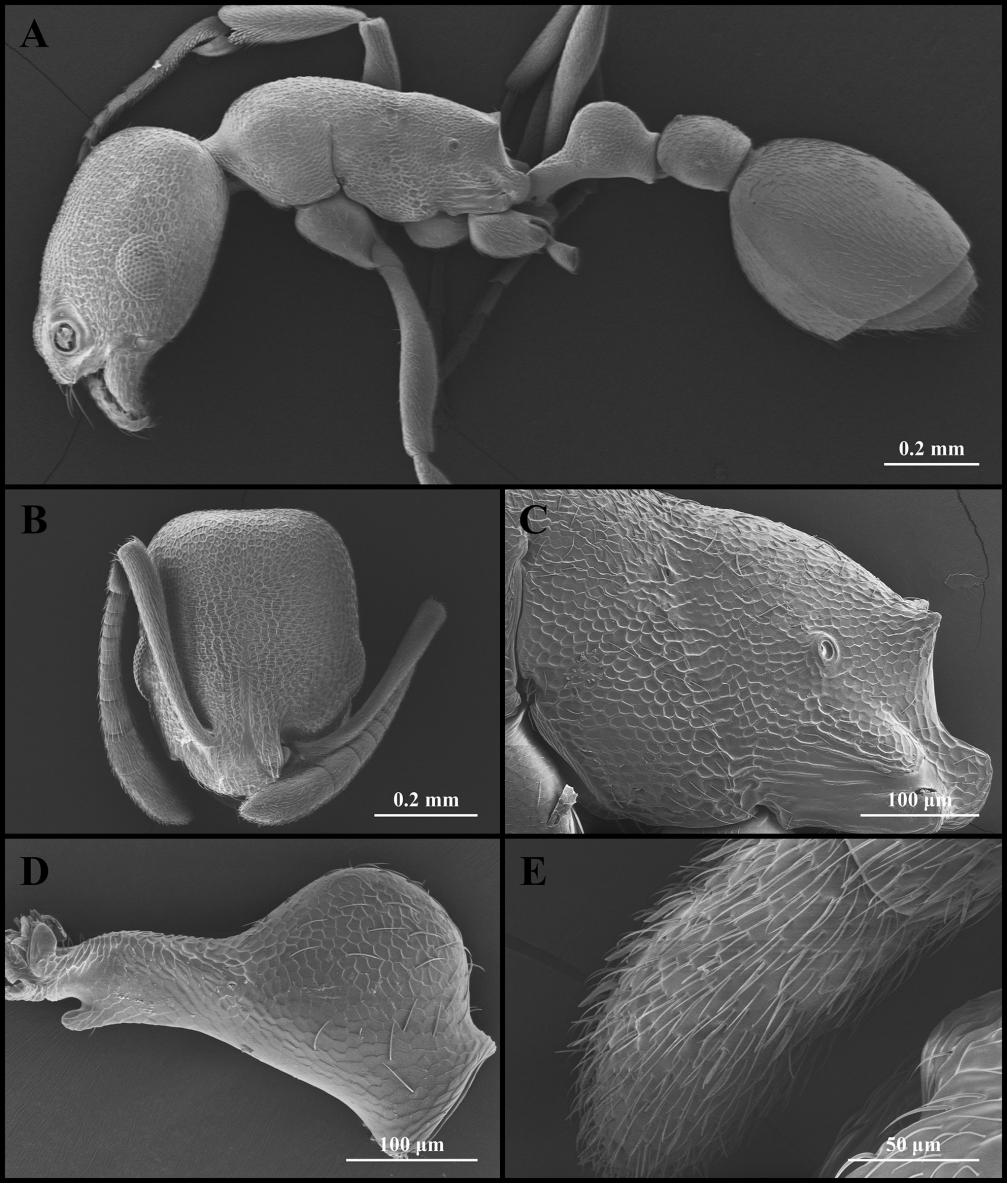
Scanning electron micrographs of *Cardiocondyla itsukii* (worker) (CAU, CAU-ANT-2520-02, imaged destructively). **A**. Body, lateral view; **B**. Head, full-face view; **C**. Propodeum, lateral view; **D**. Petiole, lateral view; **E**. Apical antennomere.

#### Non-type material examined.

**Reunion** • 1 worker, Le Port, 20°56'18"S, 55°17'41"E, 35 m elev., 17-iv-2020, Seifert et al. leg. (CASC, CASENT0125193) [images examined]. **China** • 1 worker, Yunnan Province, Jiangchuan City, 24°24'54"N, 102°50'17"E, 1705 m elev., 31-vii-2023, Yifan Fu leg. (CAU, CAU-ANT-2520-01); • 1 worker, Yunnan Province, Chengjiang City, 24°37'23"N, 102°50'51"E, 1694 m elev., 1-viii-2023, Yifan Fu leg. (CAU, CAU-ANT-2520-02).

#### Worker description.

Head longer than wide, with weakly concave or flat occipital margin and rounded lateral corners (Figs 3B, 4B). Antennae long; scape reaching the occipital margin (Figs 3B, 4B). Frontal lobes narrow (Figs 3B, 4B). Anterior clypeal margin flat (Figs 3B, 4B). Masticatory margin of mandible with five teeth. Metanotal groove present (Figs 3A, 4A). Propodeal spines short and dentate (Fig. 4C). Metapleural gland orifice slit-like (Figs 3A, 4C). Petiolar peduncle long and stout (Fig. 4A). Petiolar node long, quadrate in profile (Fig. 4D). Postpetiole compressed, with ventral process weakly developed (Fig. 4D).

***Sculpture***. Frontal area foveate, without centre striae, the foveae shallow and often confluent (Fig. 4B). Frontal lobes and clypeus striate (Fig. 4B). Antennae smooth (Fig. 4B). Mesosoma alveolate (Fig. 4A, C). Petiole and postpetiole shallow alveolate (Fig. 4D). Abdominal segments IV–VII smooth (Fig. 4A).

***Pilosity***. Body densely covered with short, appressed pubescence (Fig. 4A). Anterior clypeal margin with long, appressed hairs and three long, erect setae (Fig. 4B). Antennae bearing fine elongate hairs, and long flattened setae (Fig. 4E). Abdominal segments IV–VII with dense, long, appressed pubescence (Fig. 4A).

#### Distribution.

Guangxi (Seifert et al. 2017), Yunnan (Liu et al. 2020).

#### Remarks.

**Figure 5.**
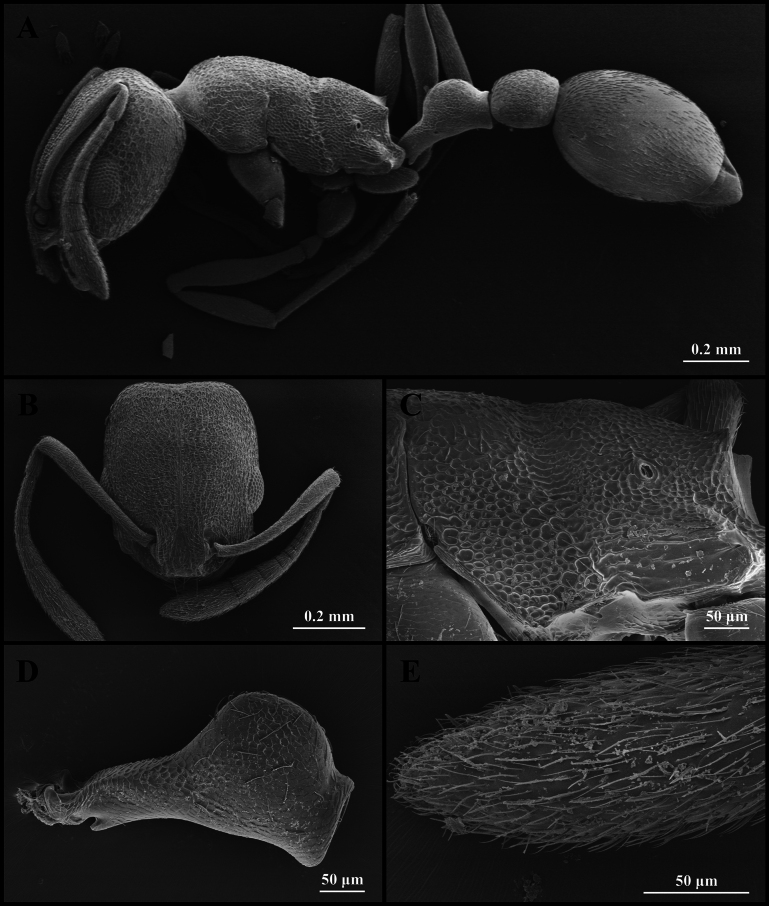
Scanning electron micrographs of *Cardiocondyla kagutsuchi* (worker) (CAU, CAU-ANT-2530-01, imaged destructively). **A**. Body, lateral view; **B**. Head, full-face view; **C**. Propodeum, lateral view; **D**. Petiole, lateral view; **E**. Apical antennomere.

The species *Cardiocondyla itsukii* was described as a new species in 2017, with documented distribution records in Guangxi (Seifert et al. 2017) and Yunnan (Liu et al. 2020), China. The species *C. itsukii* and *C. kagutsuchi* are highly similar morphologically. Accurate identification requires the use of the key in Seifert et al. (2017). However, they can be distinguished by the morphology of the petiolar node and pronotum. In lateral view, the base of the petiolar node is shorter compared to that of *C. kagutsuchi* (Figs 3A, 4D, 5D). And in *C. kagutsuchi*, the anterolateral margin of the pronotum exhibits distinct shoulders in dorsal view (Figs 3C, 4A, 5A).

### Cardiocondyla kagutsuchi

Taxon classification

Animalia

HymenopteraFormicidae

Terayama, 1999

0C32E98A-F30A-5614-905C-907725F0429B

Fig. 5

Cardiocondyla kagutsuchi Terayama, 1999: 100, figs 1–9 (worker, queen, male and ergatoid male), Japan [images examined].

#### Type material examined.

**Japan** • 1 paratype worker, Ishigaki Is., Omoto Mt., 01-vi-1988, K. Yamauchi leg. (SMNG, ANTWEB1041248) [images examined].

#### Non-type material examined.

**Fiji** • 1 worker, Viti Levu, Lautoka Port, 17°36'18"S, 177°26'24"E, 5 m elev., 15-ii-2007, E.M. Sarnat leg. (EMSC, CASENT0171071) [images examined]. **Hawaii** • 1 worker, Oahu, Waikane Trail I, 21°29'51"N, 157°52'20"W, 366 m elev., 30-ix-1989 (LACM, CASENT0103438) [images examined]. • 1 worker, Kaua’i, Koke’e State Park, Nualolo Trail, 22°12'N, 159°42'W, 04-ix-2006, J. Heinze leg. (CASC, CASENT0119698) [images examined]. • 1 worker, Mauna Ulu, Volcanoes National Park, 19°22'14"N, 155°12'15"W, 970 m elev., 01-viii-1981, P.S. Ward leg. (UCDC, CASENT0914961) [images examined]. **Indonesia** • 1 worker, Nusa Tenggara Timur, Pulau Bajo, Manggarai Barat, 08°29'19"S, 119°52'06"E, 1 m elev., 17-iv-2012, R. Sandidge leg. (EMEC, CASENT0248778) [images examined]. **Kiribati** • 1 worker, Phoenix Is., Enderbury, 07-v-2000, Okihiro leg. (AMNH, CASENT0178360) [images examined]. **Palau** • 1 worker, Sonsorol, 04°19'07"N, 132°18'37"E, 30-x-2006, J.E. Czekanski-Moir leg. (FMNH, FMNHINS0000065587) [images examined]. **Solomon Isls** • 1 worker, Guadalcanal Is., Austen Mt., Japanese Peace Memorial, 09°27'18"S, 159°58'48"E, 118 m elev., 30-i-2008, E.M. Sarnat leg. (EMSC, CASENT0219738) [images examined]. **China** • 1 worker, Yunnan Province, Jiangchuan City, 24°24'54"N, 102°50'17"E, 1705 m elev., 31-vii-2023, Yifan Fu leg. (CAU, CAU-ANT-2530-01).

#### Worker description.

Head longer than wide, with weakly concave occipital margin and rounded lateral corners (Fig. 5B). Antennae long, scapes reaching the occipital margin (Fig. 5B). Frontal lobes narrow (Fig. 5B). Anterior clypeal margin flat (Fig. 5B). Metanotal groove obvious (Fig. 5A, C). Propodeal spines short and dentate (Fig. 5A, C). Metapleural gland orifice slit-like (Fig. 5A). Petiolar peduncle long and stout. Petiolar node long, quadrate in profile (Fig. 5D). Postpetiole compressed, with ventral process weakly developed (Fig. 5D).

***Sculpture***. Frontal area centrally striate, surrounded by foveate-striate sculpture, the foveae shallow and often confluent (Fig. 5B). Frontal lobes and clypeus striate (Fig. 5B). Antennae smooth (Fig. 5B). Mesosoma alveolate (Fig. 5A, C). Petiole and postpetiole shallow alveolate (Fig. 5D). Abdominal segments IV–VII smooth (Fig. 5A).

***Pilosity***. Body densely covered with short, appressed pubescence (Fig. 5A). Anterior clypeal margin with long, appressed hairs and three long, erect setae (Fig. 5B). Antennae bearing fine elongate hairs, and long appressed setae (Fig. 5E). Abdominal segments IV–VII with dense, long, appressed pubescence (Fig. 5A).

#### Distribution.

Yunnan (Seifert 2003), Taiwan (Terayama 2009).

#### Remarks.

The species *Cardiocondyla kagutsuchi* was described by Terayama (1999) and was subsequently redescribed by Seifert (2003). The documented distribution records for this species include Yunnan Province (Seifert 2003) and Taiwan Province (Terayama 2009). This species can also be distinguished from *C. itsukii* by its longer head. *Cardiocondyla itsukii* exhibits an extreme degree of morphological similarity to *C. strigifrons* Viehmeyer, 1922. The two species are nearly indistinguishable based on any single morphological character. Consequently, reliable species separation requires multivariate statistical analysis incorporating nearly the entire set of Numeric Morphology Based Alpha-Taxonomy (NUMOBAT) characters (Seifert et al. 2017), while studies covering the distribution of this species in China often reference only prior distribution records and basic morphological examinations. Therefore, the distribution records of *C. itsukii* within China require further verification.

### Cardiocondyla nuda

Taxon classification

Animalia

HymenopteraFormicidae

(Mayr, 1866)

7EFE60B6-26D1-52F5-A353-337768FC4C51

Fig. 6

Leptothorax nudus Mayr, 1866: 508 (worker), FIJI [images examined]. Queen and ergatoid male described: Emery 1897: 588. Combined in Cardiocondyla: Forel 1881: 6.

#### Type material examined.

**Fiji** • 1 lectotype worker, Ovalau, 17°37'S, 178°10'E, Godeffroy leg. (NHMW, CASENT0919732) [images examined]. **Locality Unkno wn** • 1 syntype worker, labelled: “*nuda* G. Mayr, Type.”, “Collect. G. Mayr”, “Brit. Mus. 1922–501.”, and “BMNH(E)1014959" (BMNH, CASENT0901758) [images examined].

**Figure 6.**
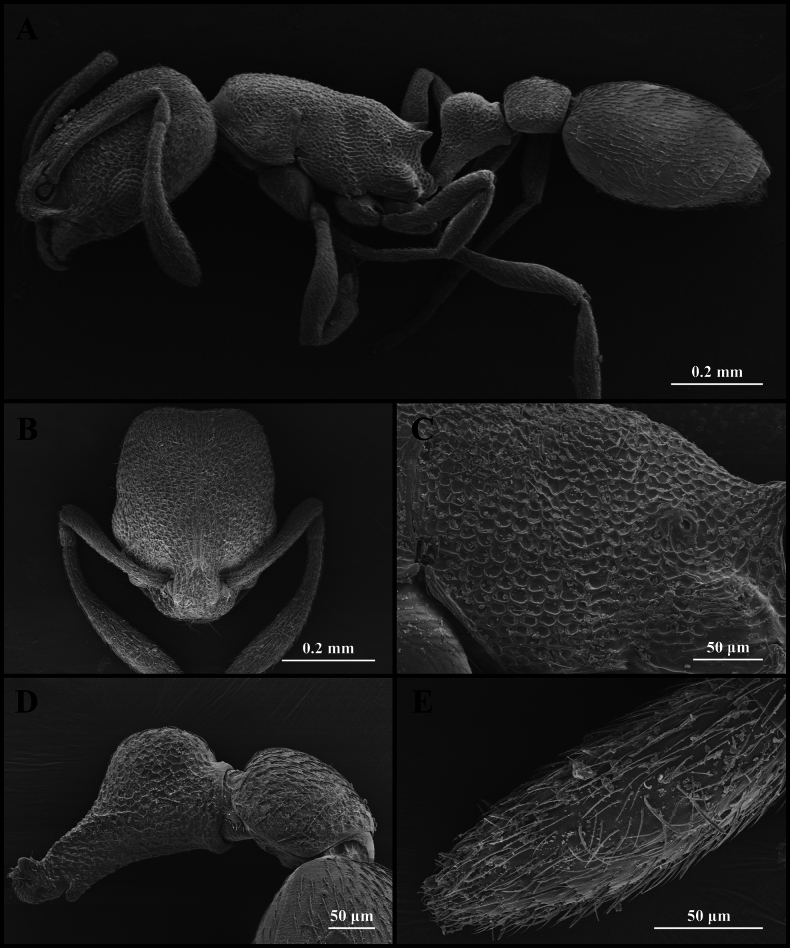
Scanning electron micrographs of *Cardiocondyla nuda* (worker) (CAU, CAU-ANT-2540-01, imaged destructively). **A**. Body, lateral view; **B**. Head, full-face view; **C**. Propodeum, lateral view; **D**. Petiole, lateral view; **E**. Apical antennomere.

#### Non-type material examined.

**Fiji** • 1 worker, Rewa, Viti Levu, at Sauniwaqa, near Nuku Village, 18°06'06"S, 178°01'08"E, 50 m elev., 10-ii-2007, E.M. Sarnat leg. (EMSC, CASENT0181806), {latitude/longitude not within Rewa, see AntWeb} [images examined]. **Micronesia** • 1 worker, Pohnpei, Sokehs Ridge, 200 m elev., 30-vii-1994, Ron Clouse leg. (AMNH, CASENT0178361) [images examined]. **Palau** • 1 worker, Sonsorol Is., 05°19'36"N, 132°13'23"E, 29-x-2006, J.E. Czekanski-Moir leg. (FMNH, FMNHINS0000051550), {latitude/longitude not within Sonsorol Is., see AntWeb} [images examined]. **China** • 1 worker, Yunnan Province, Jiangchuan City, 24°24'54"N, 102°50'17"E, 1705 m elev., 31-vii-2023, Yifan Fu leg. (CAU, CAU-ANT-2540-01).

#### Worker description.

Head longer than wide, with concave occipital margin and rounded lateral corners (Fig. 6B). Antennae short; scapes surpassing the midlength of the head but not reaching the occipital margin (Fig. 6B). Frontal lobes narrow (Fig. 6B). Anterior clypeal margin weakly emarginate (Fig. 6B). Masticatory margin of mandible with five teeth. Dorsal outline of mesosoma flat (Fig. 6A). Metanotal groove weak (Fig. 6A, C). Propodeal spines short (Fig. 6C). Metapleural gland orifice slit-like (Fig. 6A). Petiolar peduncle short and stout. Petiolar node short (Fig. 6D). Postpetiole compressed, with ventral process weakly developed (Fig. 6D).

***Sculpture***. Frontal area centrally striate, surrounded by foveate-striate sculpture, the foveae shallow and often confluent (Fig. 6B). Frontal lobes and clypeus striate (Fig. 6B). Antennae smooth (Fig. 6B). Mesosoma alveolate (Fig. 6A, C). Petiole and postpetiole alveolate (Fig. 6D). Abdominal segments IV–VII smooth (Fig. 6A).

***Pilosity***. Body densely covered with short, appressed pubescence (Fig. 6A). Anterior clypeal margin with long, appressed hairs and three long, erect setae (Fig. 6B). Antennae bearing fine elongate hairs, and long flattened setae (Fig. 6E). Abdominal segments IV–VII with dense, long, appressed pubescence (Fig. 6A).

#### Distribution.

Xizang (Huang and Zhou 2006), Qinghai (Tie and Xu 2004), Sichuan (Zhang and Zheng 2002), Yunnan (Li et al. 2007), Ningxia (Xin et al. 2011), Gansu (Tie and Xu 2004), Guangxi (Zhou 2001), Guangdong (Wang et al. 2012), Hunan (Huang and Zhou 2006), Hubei (Zhou 2001), Henan (Wang 2008), Shandong (Huang and Zhou 2006), Hainan (Guénard and Dunn 2012), Taiwan (Tsai et al. 2009), Fujian (Huang and Zhou 2006), Zhejiang (Tang et al. 1985).

#### Remarks.

The species *Cardiocondyla nuda* was described by Mayr (1866), and a lectotype was designated by Seifert et al. (2017). This species has documented distribution records from sixteen provinces in China. However, the species in the *nuda* group are extremely difficult to distinguish morphologically, these distribution records should be advised for re-examination. The petiolar peduncle of the species is shorter than the node (Fig. 6D) and it is significantly shorter than other species in the *nuda* group of China (not in FMNHINS0000051550, it is probably *C. kagutsuchi*).

##### The *sima* group, new record to China

### Cardiocondyla zhoui

Taxon classification

Animalia

HymenopteraFormicidae

Huang, Zhong, Wu, Huang & Tian 2025 sp. nov.

B50518AC-4641-50C8-AEAB-A9656AD2C8E7

https://zoobank.org/CC0B9E27-C741-4E59-8EA2-6E7E7FF026EE

Fig. 7

#### Chinese common name.

周氏心结蚁.

**Figure 7.**
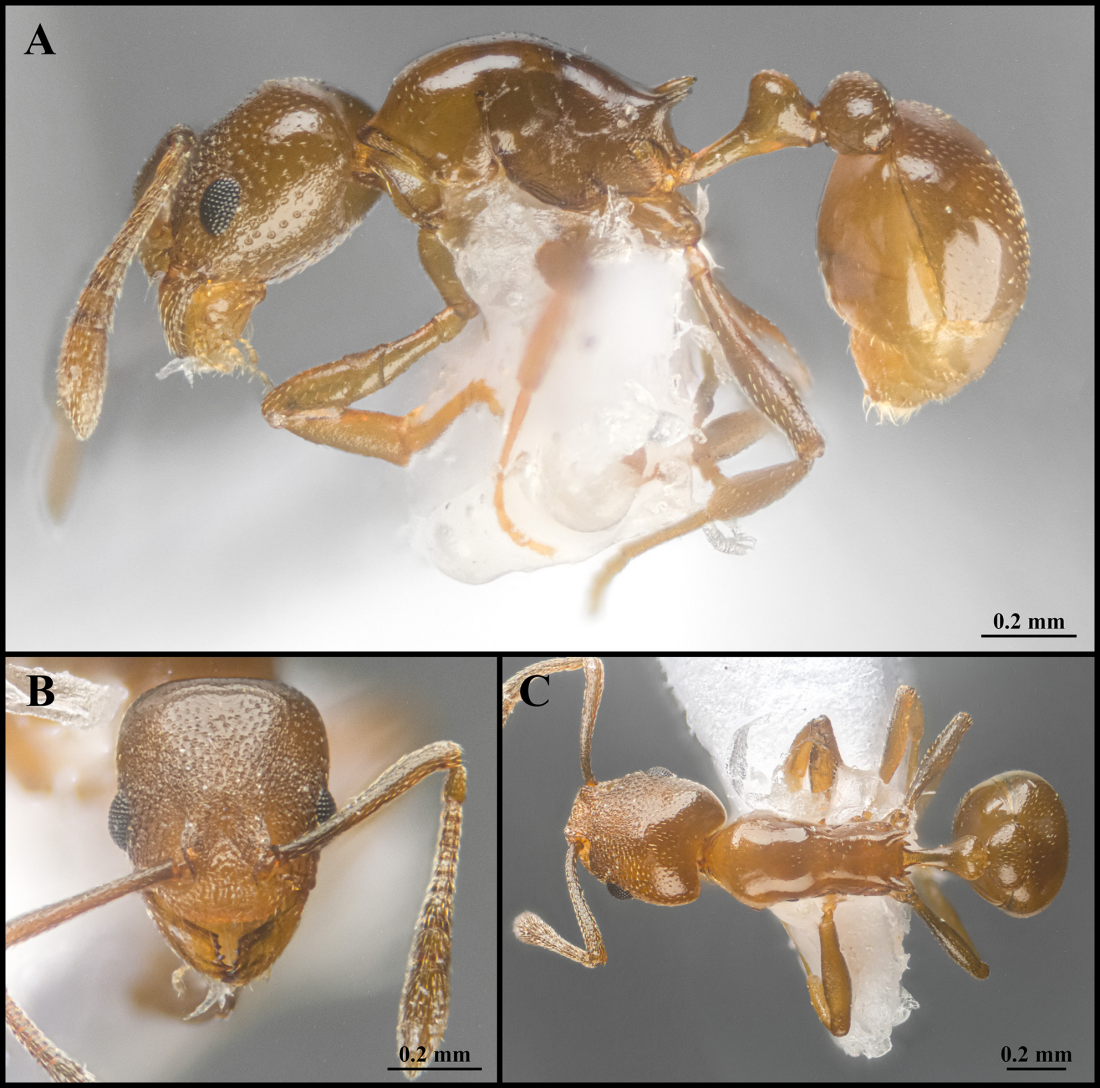
Worker of *Cardiocondyla zhoui* sp. nov. (Holotype, CAU, CAU-ANT-2550-01). **A**. Body in lateral view; **B**. Head in full-face view; **C**. Body in dorsal view.

#### Type material.

• Holotype worker, China, Yunnan Prov., Xishuangbanna, Jinghong City, near Manshanan, 21°47'07"N, 100°50'25"E, 780 m elev., 23-i-2024, Jixuan Li leg. (CAU, CAU-ANT-2550-01). • Paratypes, 4 workers, same data as holotype (CAU, CAU-ANT-2550-02, CAU-ANT-2550-03, CAU-ANT-2550-04, CAU-ANT-2550-05).

#### Diagnosis.

This species is assigned to the *sima* group of *Cardiocondyla* based on the following combination of characters: 3-segmented antennal club, elongate-oval petiolar node, body bearing only appressed pubescence (Seifert 2023a); and 11-segmented antennae, narrow petiolar peduncle, well-developed petiolar node (Seifert 2003). It is morphologically similar to *C. sima* Wheeler, 1935 from the Philippines, sharing these diagnostic traits. However, *C. zhoui* sp. nov. differs in that the occipital surface is smooth and shining, the anterior clypeal margin is evenly convex, the mesosomal outline is more elevated, and the petiolar peduncle is more elongated.

#### Description of holotype worker.

Measurements (mm) and indices. EL 0.14, FRW 0.17, HL 0.54, HW 0.45, ML 0.68, PH 0.17, PL 0.31, PPH 0.16, PPL 0.16, PPW 0.24, PRW 0.35, PSL 0.09, PW 0.16, SL 0.47, CI 84, SI 103, PI 50, PHI 90, PPI 151, PPHI 154, WI 156.

***Head*** (Fig. 7B). Subquadrate, longer than wide; occipital margin weakly convex, lateral margins almost straight, gradually and slightly converging anteriorly; occipital corners blunt, with rounded transitions. Eye located at midline of head, distinctly convex, breaking lateral head margin. Frontal lobe broad, covering antennal foramen. Supraclypeal area wide. Antennae 11-segmented; scape long, surpassing occipital margin by ~1/5 of its length; last three segments forming a distinct club, apical funicular segment dilated. Clypeus broad, its anteromedian margin evenly convex, inserted broadly between frontal lobes. Mandibles triangular; masticatory margin with five teeth.

***Mesosoma*** (Fig. 7A, C). In lateral view, promesonotal dorsum evenly convex; promesonotal suture absent. Metanotal groove absent. Mesopropodeal suture absent. Propodeal dorsum and declivity sloping, dorsum slightly longer than declivity; propodeal spines long, acute, straight, and directed posteriorly; propodeal lobe produced backward as a blunt tooth. In dorsal view, pronotum distinctly wider than mesonotum and propodeum; propodeum constricted anteriorly.

***Metasoma*** (Fig. 7A, C). Petiole in lateral view with a long, slender peduncle occupying ~ 1/3 of total petiolar length; anterior face nearly vertical, weakly concave; anterodorsal corner blunt, forming a rounded-rectangular transition; dorsal outline weakly convex and rounded; ventral margin straight. Postpetiole in lateral view circular, as high as petiole; in dorsal view elongate-oval, distinctly broad, and wider than petiole. Abdominal segments IV–VII oval in outline. Abdominal segment IV is longer than half length of abdominal segments V–VII and wider than abdominal segment IV.

***Sculpture*** (Fig. 7). Frontal area rugulose-foveate, occiput smooth with comparatively sparse foveae. Gena with sparse, longitudinal striae. Frontal lobes and clypeus rugulose. Mesosoma smooth overall; pronotum and mesonotum smooth and shining. Propodeum smooth to finely imbricate, metapleuron with weak, longitudinal striation. Petiole and postpetiole smooth to finely imbricate. Abdominal segments IV–VII entirely smooth.

***Pilosity*** (Fig. 7). Body sparsely covered with short, appressed pubescence. Antennae and mandibles with more dense, short, appressed pubescence. Anterior clypeal margin with long, appressed hairs and three long, erect setae. Mesosoma and legs with sparse, extremely short, appressed pubescence. Postpetiole and abdominal tergite IV with extremely dense, short, appressed pubescence. Abdominal segments IV–VII with dense, short, appressed pubescence.

***Colour***. Whole body yellow-brown.

#### Measurements (mm) and indices of paratypes (n = 4).

EL 0.13–0.14, FRW 0.18–0.19, HL 0.52–0.57, HW 0.44–0.46, ML 0.66–0.70, PH 0.17–0.18, PL 0.28–0.30, PPH 0.16–0.18, PPL 0.17–0.18, PPW 0.23–0.25, PRW 0.34–0.36, PSL 0.08, PW 0.15–0.16, SL 0.45–0.48, CI 81–86, SI 100–104, PI 53–56, PHI 84–94, PPI 136–144, PPHI 138–147, WI 149–159.

#### Other castes.

Unknown.

#### Distribution.

Only known in Yunnan, China.

#### Biology.

Nests of this species were found within the crown structure of nests of *Tetramorium wroughtonii* Forel, 1902. However, the nature of the association between the two species remains unclear.

#### Etymology.

The specific epithet zhoui is a genitive noun, named in honour of the late Professor Shanyi Zhou, a pioneering Chinese myrmecologist, in recognition of his pioneering work in the field of Chinese myrmecology.

##### The *stambuloffii* group

### Cardiocondyla gibbosa

Taxon classification

Animalia

HymenopteraFormicidae

Kuznetsov-Ugamsky, 1927

BEE662E5-BBA4-5408-ACF0-752B7AFF0E61

Cardiocondyla elegans subsp. gibbosa Kuznetsov-Ugamsky, 1927: 37, figs 1–15 (worker), Kazakhstan [images examined]. Synonymised with C. koshewnikovi Ruzsky, 1902: 480: Dlussky et al. 1990: 195. Raised to species: Seifert 2003: 268.

#### Type material examined.

**Kazakhstan** • 1 lectotype worker, Perovsk (Kizil-Orda), Suzak, 45°N, 70°E, 3-vii-1923 (31-iii-1923?), N.N. Kusnetzov-Ugamsky leg. (NHMB, CASENT0912878) [images examined].

#### Distribution.

Xinjiang (Gulzar 2014).

#### Remarks.

The species *Cardiocondyla gibbosa* was described by Kuznetsov-Ugamsky (1927). Later this species was once considered as a subspecies of *C. elegans* Emery, 1869 (Tarbinsky 1976), but was later treated as a junior synonym of *C. koshewnikovi* Ruzsky, 1902 (Dlussky et al. 1990; Radchenko 1995). This species was redescribed and a lectotype was designated by Seifert (2003), but later, Seifert (2023b) redundantly designated a lectotype and two paralectotypes. The collection date of the lectotype is doubtful. The date on the original label is closer to 31 July than to 3 July as recorded in Seifert (2003), presumably representing an error. This species has documented distribution records only from Xinjiang in China (Gulzar 2014). The species *C. gibbosa* is similar to *C. koshewnikovi*, and the two species are quite difficult to distinguish. It can be distinguished from other known species by the strong and sparse longitudinal carinae on the malar areas, which extend over the eyes. Additionally, the two species can be distinguished by the much longer pubescence and the more elongate head of *C. gibbosa* compared to *C. koshewnikovi* Kuznetsov-Ugamsky, 1927 (Seifert 2023b).

### Cardiocondyla koshewnikovi

Taxon classification

Animalia

HymenopteraFormicidae

Ruzsky, 1902

2CC883B1-8BCC-5FE3-BB22-06C2CF2E8AF3

Cardiocondyla koshewnikovi Ruzsky, 1902: 480 (worker and queen), Kazakhstan [images examined]. Synonymised with C. stambuloffii Forel, 1892: Pisarski 1970: 308. Raised to species: Dlussky et al. 1990: 195.

#### Type material examined.

**Kazakhstan** • 1 lectotype worker, Aral Sea, Syr Darya, 31-xii-1902, M. R. leg. (M. Ruzsky leg.?) (MHNG, CASENT0908348) [images examined]. • 1 lectotype (?) worker, Aral Sea, 46°48'N, 61°40'E (MSNG, CASENT0904464) [images examined].

#### Non-type material examined.

**Kazakhstan** • 1 worker, W-Ufer des Sassy-Kol, 46°41'57"N, 80°35'00"E, 358 m elev., 07-viii-2001, Seifert leg. (SMNG, ANTWEB1048517) [images examined].

#### Distribution.

Xinjiang (Seifert 2003).

#### Remarks.

The species *Cardiocondyla koshewnikovi* was described by Ruzsky (1902). It was historically considered either a synonym or a subspecies of *C. stambuloffii* Forel, 1892 (Forel 1902; Pisarski 1970). Dlussky (1990) raised *C. koshewnikovi* to species rank based on morphological differences observed in males. This species is now regarded as a sister species to *C. stambuloffii* and is primarily distributed in Central Asia (Seifert 2003). Seifert (2003) designated a lectotype (CASENT0904464) and provided a redescription. Specimen CASENT0904464 lacks both collector and collection date information. In China, *C. koshewnikovi* has documented distribution records only from Xinjiang (Seifert 2003). It is noteworthy that due to the morphological similarity between *C. koshewnikovi* and *C. gibbosa*, it is highly probable that records of *C. gibbosa* from Xinjiang actually represent misidentifications of *C. koshewnikovi*. This species can be distinguished from other known species by the morphology of its petiolar node and peduncle. The anterior junction of petiolar node and peduncle exhibits a strongly, steeply sloping profile.

### Cardiocondyla rolandi

Taxon classification

Animalia

HymenopteraFormicidae

Seifert, 2023b

D637CE02-C520-52D3-91D2-E8616D1A9932

Cardiocondyla rolandi Seifert, 2023b: 51, figs 94–97 (worker, queen, and male), China [images examined].

#### Type material examined.

**China** • 1 worker, Xinjiang, Tarim Basin, edge of oasis Yengisar, 41°58'26"N, 84°29'26"E, 1038 m elev., 03-ix-2004, Schultz leg. (SMNG, ANTWEB1048533) [images examined].

#### Distribution.

Xinjiang (Seifert 2023b).

#### Remarks.

The species *Cardiocondyla rolandi* was described by Seifert (2023b). Its type locality is located in Xinjiang, China. Morphologically, *C. rolandi* can be distinguished from other species by its convex clypeus and the morphology of the petiolar node. The anterior junction of the petiolar node and peduncle is more gentle, and not obviously steep. Moreover, its propodeal spines project obliquely dorsally in lateral view.

### Cardiocondyla stambuloffii

Taxon classification

Animalia

HymenopteraFormicidae

Forel, 1892

A14F7116-33EE-5BED-8144-6A813AC4606E

Fig. 8

Cardiocondyla stambuloffii Forel, 1892: 310, pl. 5, fig. 1 (worker, queen and ergatoid male), Bulgaria [images examined]. Ergatoid male described: Kugler 1984: 8.
Cardiocondyla bogdanovi Ruzsky, 1905: 630, figs 155, 157, 158 (worker and queen), Armenia [images examined]. Synonymised: Seifert 2003: 262.
Cardiocondyla montandoni Santschi, 1912: 657, figs 1, 3 (worker), Romania [images examined]. Synonymised: Pisarski 1962: 332.
Cardiocondyla stambuloffii subsp. *taurica* Karavaiev, 1927: 288, fig. 2 (worker), Ukraine [images examined]. Synonymised: Arnol’di and Dlussky 1978: 538.

#### Type material examined.

**Bulgaria** • 19 syntype workers, Anchialo, 42°33'32"N, 27°38'38"E (BMNH, CASENT0901756; MSNG, CASENT0904463; MHNG, CASENT0908347; DEI, FOCOL0296-1, FOCOL0296-2, FOCOL0296-3, FOCOL0297-1, FOCOL0297-2; MNHU, FOCOL1608, FOCOL1609, FOCOL1610, FOCOL2904, FOCOL2905, FOCOL2906; SMNK, FOCOL0693-1, FOCOL0693-2; NMOK, FOCOL0783-1, FOCOL0783-2, FOCOL0784) [images examined]. • 1 syntype worker, Burgas, 42°30'N, 27°28'E (SIZK, CASENT0917797) [images examined].

**Figure 8.**
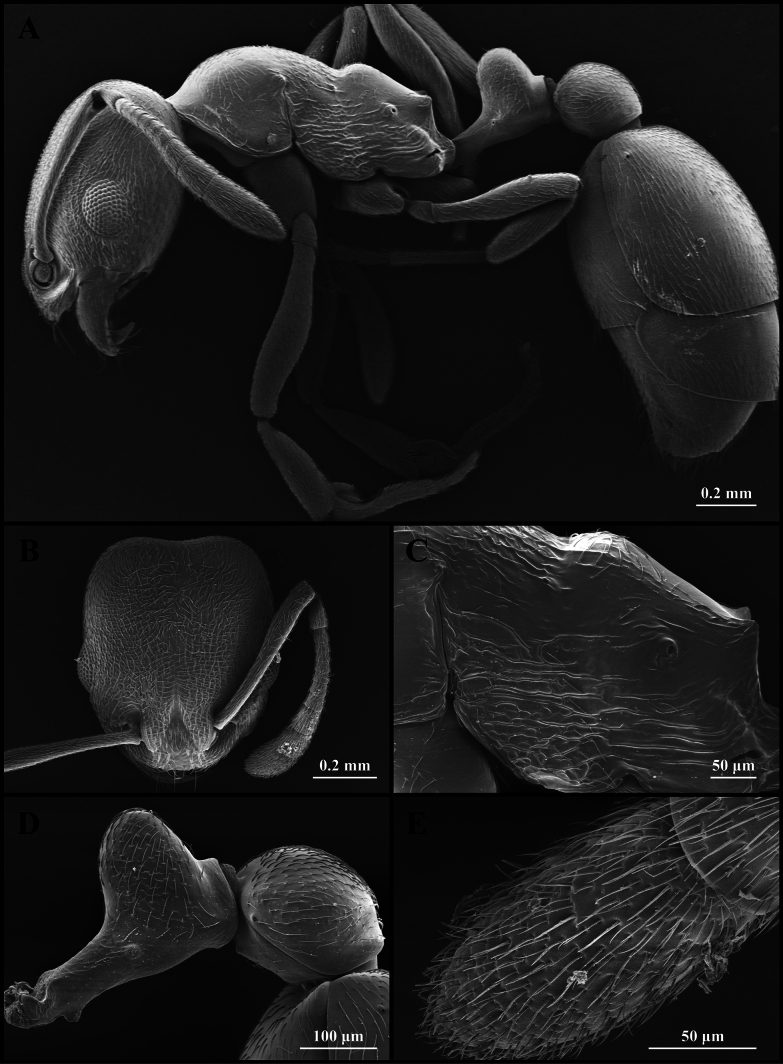
Scanning electron micrographs of *Cardiocondyla stambuloffii* (worker) (CAU, CAU-ANT-2560-01, imaged destructively). **A**. Body, lateral view; **B**. Head, full-face view; **C**. Propodeum, lateral view; **D**. Petiole, lateral view; **E**. Apical antennomere.

#### Non-type material examined.

**Georgia** • 1 worker, Samtskhe-Javakheti, bank of river Podshori, 41°38'57"N, 42°51'50"E, 1021 m elev., 27-viii-2010, Heinze leg. (SMNG, ANTWEB1048539) [images examined]. **China** • 1 worker, Inner Mongolia, Wuhai City, 31-v-2025, Xuanhao Cheng leg. (CAU, CAU-ANT-2560-01).

#### Worker description.

Head as long as wide, with strongly concave occipital margin and rounded lateral corners (Fig. 8B). Antennae long; scapes reaching the occipital margin (Fig. 8B). Frontal lobes broad (Fig. 8B). Anterior clypeal margin flat or convex (Fig. 8B). Metanotal groove strongly concave (Fig. 8A, C). Propodeum strongly convex anterodorsally (Fig. 8C). Propodeal spines blunt and dentate (Fig. 8C). Metapleural gland orifice long slit-like (Fig. 8A). Petiolar peduncle shallow (Fig. 8A). Petiolar node tall, as long as peduncle (Fig. 8D). Postpetiole oval, anteroventral corner dentate (Fig. 8D).

***Sculpture***. Frontal area costate sculpture, costae sinuous; occiput smooth (Fig. 8B). Frontal lobes. Clypeus smooth centrally, striate laterally (Fig. 8B). Antennae smooth (Fig. 8B). Pronotum smooth; mesopleuron with mixed costae and reticulation (Fig. 8A). Propodeal lobe reticulate, remainder of propodeum smooth (Fig. 8C). Metapleuron striate. Petiole and postpetiole finely imbricate to smooth (Fig. 8D). Abdominal segments IV–VII smooth (Fig. 8A).

***Pilosity***. Body densely covered with short, appressed pubescence (Fig. 8A). Anterior clypeal margin with long, appressed hairs and three long, erect setae (Fig. 8B). Antennae bearing fine elongate hairs, and long flattened setae (Fig. 8E). Abdominal segments IV–VII with extremely dense, long, appressed pubescence (Fig. 8A).

#### Distribution.

Xinjiang (Collingwood and Heatwole 2000), Inner Mongolia (new record).

#### Remarks.

The species *Cardiocondyla stambuloffii* was described by Forel (1892). Like most species in its species group, it has documented records from Xinjiang, China (Collingwood and Heatwole 2000). Species within the *stambuloffii* group are difficult to distinguish from one another. Accurate differentiation requires the key in Seifert (2023b) and relies on precise morphometric measurements. Within the *stambuloffii* group, *C. stambuloffii* can be distinguished by the presence of a transverse carinae on the lateral sides of the mesopleuron and propodeum anterior to the promesonotal suture. This carina is not spanned but instead is fused to the meso-metapleural suture (Fig. 8C). *Cardiocondyla stambuloffii* and *C. koshewnikovi* are characterised by a straight or slightly concave anterior labral margin, in contrast to the convex anterior labral margin (Fig. 8B) found in other species within the *stambuloffii* group.

### Cardiocondyla tibetana

Taxon classification

Animalia

HymenopteraFormicidae

Seifert, 2003

22BD93E0-FD53-5F64-A3C5-EDEDA614CF10

Cardiocondyla tibetana Seifert, 2003: 269, fig. 49 (worker), CHINA [images examined].

#### Type material examined.

**China** • Holotype and 1 paratype workers, Xinjiang, Tarim Basin, Cele National Field Research Station, 37°00'57"N, 80°43'45"E, 26-viii-1966, H. Heatwole leg. (SMNG, ANTWEB1041252, ANTWEB1048543) [images examined]. • 1 paratype worker, Tibet, 32°N, 90°E (NHMW, CASENT0919737) [images examined].

#### Distribution.

Xinjiang (Seifert 2003).

#### Remarks.

The species *Cardiocondyla tibetana* was described by Seifert (2003) and was redescribed by Seifert (2023b). Although bearing the specific epithet “tibetana”, the type specimens of *C. tibetana* are presumed to originate exclusively from Xinjiang, China, and this species has documented records only from Xinjiang. This species can be distinguished from other species by its frontal area, which is predominantly smooth with longitudinal striations and does not form a complex reticular sculpture, as well as by the lateral sides of its pronotum are largely smooth and essentially lack both punctures and carinae.

##### The *ulianini* group

### Cardiocondyla ulianini

Taxon classification

Animalia

HymenopteraFormicidae

Emery, 1889

2D6369FF-7ECC-5134-B19E-2978AE4EC0D9

Cardiocondyla elegans var. *ulianini* Emery, 1889: 441 (worker), “Turkestan” [images examined]. Queen described: Karavaiev 1911: 57. Male and ergatoids described: Marikovsky and Yakushkin 1974: 57. Initially raised to species: Agosti and Collingwood 1987: 56. Synonymised with C. elegans: Dlussky et al. 1990: 194. Re-raised to species: Seifert 2003: 229.
Cardiocondyla elegans subsp. *schkaffi* Alpatov and Arnol’di, 1928: 724 (worker and queen), Ukraine [not examined]. Synonymised: Radchenko 2016: 235.

#### Type material examined.

**“Turkestan”** • 1 lectotype worker, 45°N, 70°E, Fedschenko leg. (MSNG, CASENT0904461), {the dorsal view is another specimen?} [images examined].

#### Non-type material examined.

**Iran** • 1 worker, Miankaleh, viii-2004, O. Paknia leg. (ZMGU, ZMGU1418) [images examined]. **Kyrgyzstan** • 1 worker, Chüy, Flussterrasse 1 m über Hoshwasserlinie, 42°44'41"N, 75°49'50"E, 1230 m elev., 30-vii-2000 (SMNG, ANTWEB1048545) [images examined].

#### Distribution.

Xinjiang (Seifert 2003).

#### Remarks.

The species *Cardiocondyla ulianini* was described by Emery (1889) as a variety of *C. elegans*. It was raised to species rank and redescribed by Seifert (2003). This species has documented distribution records only from Xinjiang in China. This species can be distinguished from *C. elegans* by both its smaller vertex foveolae and several morphometric differences (Seifert 2003). The dorsal view image of the holotype specimen CASENT0904461 on AntWeb (2026) clearly represents a different specimen.

##### The *wroughtonii* group

### Cardiocondyla obscurior

Taxon classification

Animalia

HymenopteraFormicidae

Wheeler, 1929

85D64CD8-D07D-5E08-9497-6B3ED795EC01

Fig. 9

Cardiocondyla wroughtonii var. obscurior Wheeler, 1929: 44 (worker and queen), China [not examined]. Synonymised with C. wroughtonii Forel, 1890: Lin and Wu 2003: 63. Raised to species: Seifert 2003: 271.
Cardiocondyla bicolor Donisthorpe, 1930: 366 (worker), Israel [images examined]. Synonymised: Seifert 2003: 271.

#### Non-type material examined.

**Brazil** • 1 worker, Bahia, 14°48'S, 39°16'W, 16-xii-2003, J.H.C. Delabie leg. (CASC, CASENT0119909) [images examined]. **British Virgin Isls** • 1 worker, Guana Is., White Beach, 18°28'42"N, 64°34'30"W, 01-vii-1993 to 07-vii-1993, R.R. Snelling leg. (LACM, CASENT0103430) [images examined]. **California** • 1 worker, Los Angeles, Atwater, LA River, 34°06'25"N, 118°14'46"W, 110 m ± 10 m elev., 17-xi-2015 to 02-x-2016, Kelsey Baily leg. (DBBC, CASENT0799216) [images examined]. **Ecuador** • 1 worker, Galapagos Is., Santa Cruz, Mina Granillo Roja, 00°36'55"S, 90°22'01"W, 580 m elev., 19-xi-2012 (SMNG, ANTWEB1048527) [images examined]. **Fiji** • 1 worker, Nanui-i-Ra Is., Yasawa Prov., McDonald’s Resort, 17°17'52"S, 178°13'01"E, 10 m elev., 23-i-2005, E.M. Sarnat leg. (EMSC, CASENT0171038) [images examined]. **Florida** • 1 worker, Volusia, Deland, 29°02'N, 81°18'W, 10-x-1997, Stephen Deyrup leg. (LACM, CASENT0103429) [images examined]. • 1 worker, Orange, Wekiva Sprs. Park, 28°43'N, 81°27'W, 17-ii-1998, M. Deyrup and Z. Prusak leg. (ABS, CASENT0103752) [images examined]. **Micronesia** • 1 worker, Pohnpei, Quarter mile up river from Mahnd, 06°51'N, 158°10'E, 29-x-1994, Ron Clouse leg. (AMNH, CASENT0178363) [images examined]. **Saudi Arabia** • 1 worker, Al Bahah, Baljurashi Forest, 19°48'20"N, 41°42'43"E, ± 50 m, 1930 m elev., 21-ix-2011, F.A. Esteves leg. (CASC, CASENT0264663) [images examined]. **Seychelles** • 1 worker, Silhouette Is., La Passe, 04°29'05"S, 55°15'03"E, 35 m elev., 23-i-2010, B.L. Fisher et al. leg. (CASC, CASENT0217233) [images examined]. **Venezuela** • 1 worker, Aragua, Rancho Grande, PN Henri Pittier, 10°21'00"N, 67°41'06"W, 1100 m elev., 12-viii-2008, P.S. Ward leg. (PSWC, CASENT0914967) [images examined]. **China** • 1 worker, Yunnan Province, Jiangchuan City, 24°24'54"N, 102°50'17"E, 1705 m elev., 31-vii-2023, Yifan Fu leg. (CAU, CAU-ANT-2570-01).

**Figure 9.**
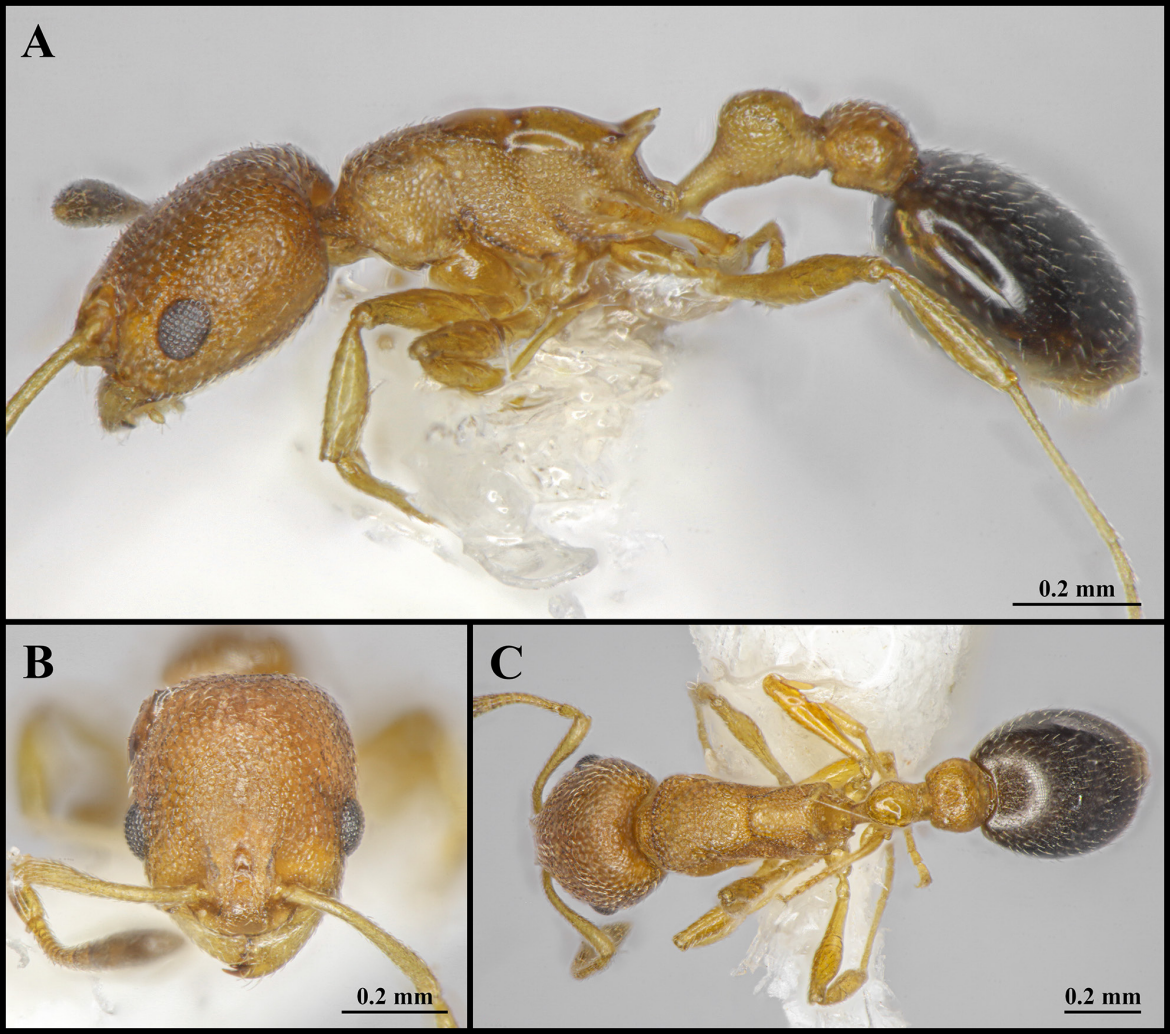
Worker of *Cardiocondyla obscurior* (CAU, CAU-ANT-2570-01, imaged destructively). **A**. Body in lateral view; **B**. Head in full-face view; **C**. Body in dorsal view.

#### Distribution.

Taiwan (Seifert, 2003), Guangxi (Hua 2006), Yunnan (Hua 2006).

#### Remarks.

**Figure 10.**
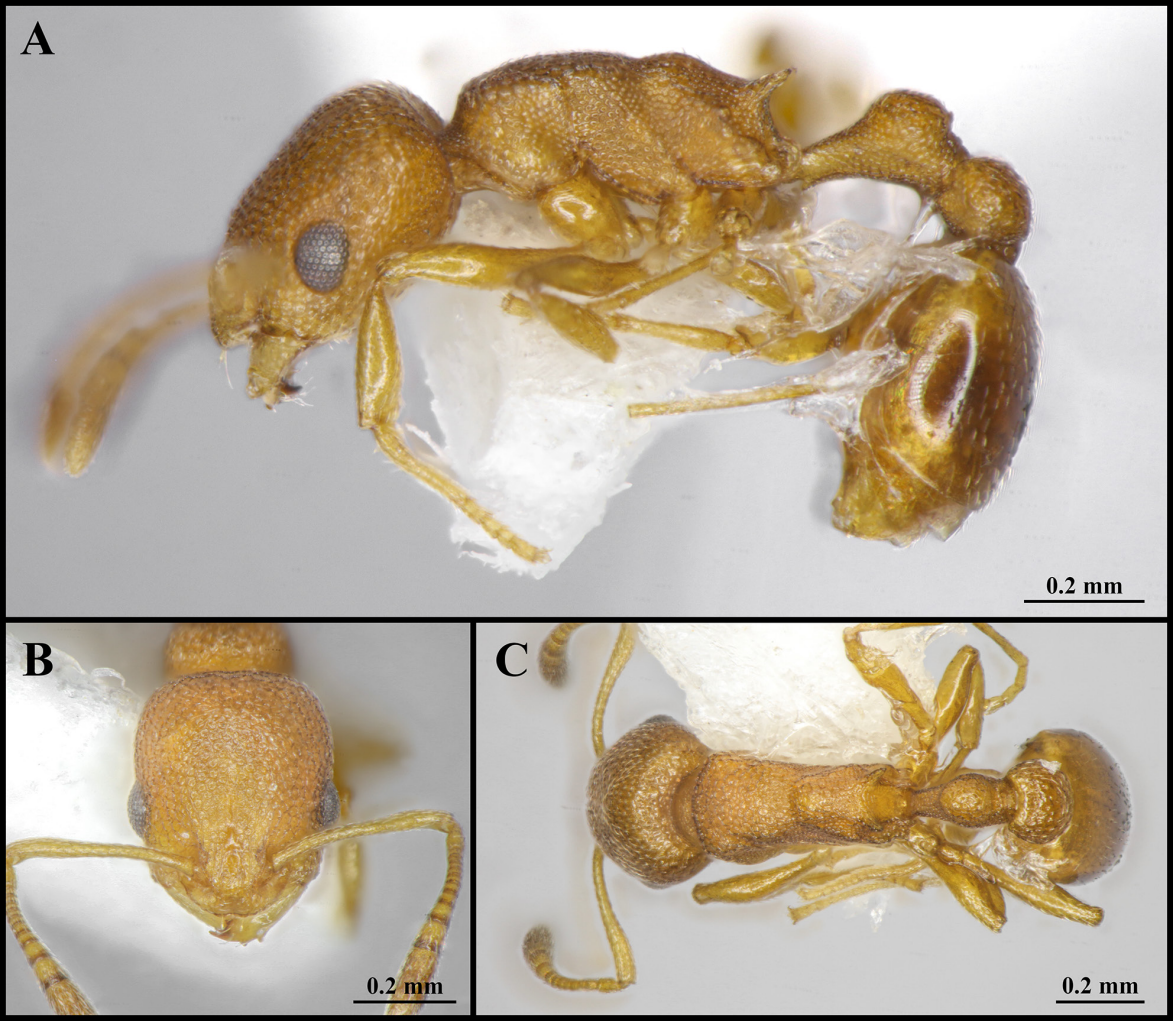
Worker of *Cardiocondyla wroughtonii* (CAU, CAU-ANT-2580-01). **A**. Body in lateral view; **B**. Head in full-face view; **C**. Body in dorsal view.

The species *Cardiocondyla obscurior* was described by Wheeler (1929) as a variety of *C. wroughtonii*. It was raised to species rank by Seifert (2003) as the senior synonym of *C. bicolor*. This species has documented distribution records in Taiwan (Seifert, 2003), Guangxi, and Yunnan (Hua 2006). However, the accuracy of the records from Guangxi and Yunnan requires further verification. The two species *C. obscurior* and *C. wroughtonii* are extremely difficult to distinguish morphologically and can only be tentatively separated by the coloration of abdominal segments IV–VII (Seifert 2003). The darker coloration of abdominal segments IV–VII of *C. obscurior* distinguishes it from *C. wroughtonii* (Figs 9A, 10A). However, Seifert (2024) proposed that the morphometric data for these two species show inconsistency with the coloration of abdominal segments IV–VII. Accurate differentiation between these two species still requires the use of the key provided in Seifert (2024) and relies on precise morphometric measurements. Both species can be distinguished from other species by their long and thin propodeal spines.

### Cardiocondyla wroughtonii

Taxon classification

Animalia

HymenopteraFormicidae

(Forel, 1890)

73EC39EC-F881-5D3F-86C7-8661117EA4B5

Figs 10, 11

Emeryia wroughtonii Forel, 1890: cxi (ergatoid male), India [images examined]. Worker and queen described: Forel 1903: 689. Male described: Kugler 1984: 7. Combined in Cardiocondyla: Forel 1892: 461.
Cardiocondyla wroughtonii var. *hawaiensis* Forel, 1899: 119 (worker) Hawaii [images examined]. Synonymised: Wilson and Taylor 1967: 56.
Cardiocondyla wroughtonii subsp. *quadraticeps* Forel, 1912: 57 (worker) Singapore [not examined]. Synonymised: Seifert 2003: 269.
Cardiocondyla wroughtonii var. *bimaculata* Wheeler, 1929: 43 (worker and queen) China [not examined]. Synonymised: Smith 1979: 1376.
Cardiocondyla emeryi subsp. *chlorotica* Menozzi, 1930: 84 (worker and queen), Somalia [not examined]. Synonymised: Bolton 1982: 317.
Cardiocondyla longispina Karavaiev, 1935: 88, fig. 14 (worker) Indonesia [images examined]. Synonymised: Seifert 2003: 269.
Cardiocondyla yamauchii Terayama, 1999: 104, figs 14–19 (worker, queen, male, and ergatoid male) Japan, Singapore [images examined]. Synonymised: Seifert 2003: 269.

#### Type material examined.

**India** • 2 syntype workers and 1 syntype (holotype) ergatoid male, Poona, 18°30'48"N, 73°51'00"E, Wroughton leg. (BMNH, CASENT0901753; MHNG, CASENT0908350, CASENT0908349) [images examined].

**Figure 11.**
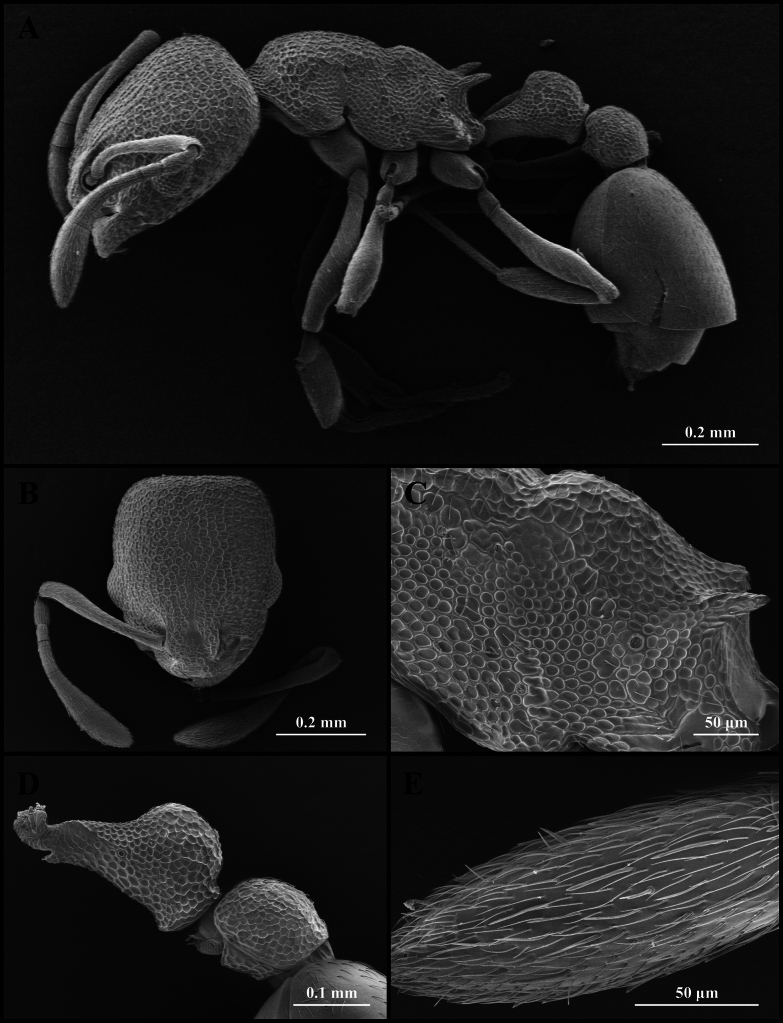
Scanning electron micrographs of *Cardiocondyla wroughtonii* (worker) (CAU, CAU-ANT-2580-02, imaged destructively). **A**. Body, lateral view; **B**. Head, full-face view; **C**. Propodeum, lateral view; **D**. Petiole, lateral view; **E**. Apical antennomere.

#### Non-type material examined.

**California** • 1 worker, Los Angeles, 34°06'18"N, 118°14'35"W, 101 m elev., 16-ii-2016, Kelsey Baily and Lisa Gonzales leg. (DBBC, CASENT0799259) [images examined]. **Florida** • 1 worker, Brevard, Palm Bay; Peckham St. NE, 28°02'N, 80°40'W, 07-viii-2000, Z. Prusak leg. (CASC, CASENT0009246) [images examined]. • 1 worker, Dade, Coral Gables, Matheson Hammock, 25°41'N, 80°16'W, 21-iv-1993, Deyrup and Ferster leg. (ABS, CASENT0103750) [images examined]. **Hawaii** • 1 worker, N Kohala Dist., W Honokane Iki R., 20°09'30"N, 155°43'40"W, 650 m elev., 29-vii-1981, P.S. Ward leg. (PSWC, CASENT0171166) [images examined]. **Israel** • 1 worker, in shipment, 22-xi-1964, R. Hashimoto leg. (USNM, CASENT0246602) [images examined]. **Kenya** • 1 worker, Kajiado, 02°28'06"S, 37°19'58"E, 01-x-1999, J. Saiboku leg. (BMNH, CASENT0102969) [images examined]. **Madagascar** • 1 worker, Mahajanga Prov., P.N. Tsingy de Bemaraha, 3.4 km 93°E Bekopaka, 19°08'31"S, 44°49'41"E, 50 m elev., 06-xi-2001 to 10-xi-2001, B.L. Fisher et al. (CASC, CASENT0474550) [images examined]. **Malaysia** • 1 worker, Sarawak, Lundu, Stadt, 01°40'30"N, 109°50'42"E, 16 m elev., 27-v-2007 (SMNG, ANTWEB1048547) [images examined]. **Mauritius** • 1 worker, Brise Mt., Bambous, 20°20'44"S, 57°45'17"E, 200 m elev., 27-v-2005, B.L. Fisher et al. (CASC, CASENT0060052) [images examined]. **Mayotte** • 1 worker, Benara Mt., 12°52'33"S, 45°09'24"E, 425 m elev., 30-xi-2007 to 02-xii-2007, B.L. Fisher et al. (CASC, CASENT0133506) [images examined]. **Moorea** • 1 worker, near Haapiti coast road km 24 S, 17°32'S, 149°54'W, 27-ii-1991, L. Morrison leg. (PSWC, CASENT0914972) [images examined]. **Oman** • 1 worker, Dhofar, Ayn Hamran, 17°05'58"N, 54°17'03"E, 106 m elev., 20-xi-2017, M.R. Sharaf leg. (KSMA, CASENT0922871) [images examined]. **China** • 2 workers, Yunnan Province, Chengjiang City, 24°33'55"N, 102°50'37"E, 1727 m elev., 3-viii-2023, Fu Yifan leg. CAU, CAU-ANT-2580-01, CAU-ANT-2580-02).

#### Worker description.

Head longer than wide, with flat occipital margin and rounded lateral corners (Figs 10B, 11B). Antennae short; scapes surpassing the midlength of the head but not reaching the occipital margin (Figs 10B, 11B). Frontal lobes broad (Figs 10B, 11B). Anterior clypeal margin weakly emarginate (Figs 10B, 11B). Metanotal groove strongly concave (Figs 10A, 11A, 11C). Propodeum strongly convex anterodorsally (Figs 10A, 11C). Propodeal spines long and sharp (Fig. 11C). Metapleural gland orifice slit-like (Fig. 11A). Petiolar peduncle short and stout. Petiolar node short (Fig. 11D). Postpetiole oval, with developed ventral process, anteroventral corner blunt (Fig. 11D).

***Sculpture***. Frontal area foveate, the foveae shallow and often confluent (Fig. 11B). Frontal lobes foveate (Fig. 11B). Clypeus rugulose (Fig. 11B). Antennae smooth (Fig. 11B). Mesosoma alveolate (Fig. 11A, C). Petiole and postpetiole alveolate (Fig. 11D). Abdominal segments IV–VII smooth (Fig. 11A).

***Pilosity***. Body densely covered with short, appressed pubescence (Fig. 11A). Anterior clypeal margin with long, appressed hairs and three long, erect setae (Fig. 11B). Antennae bearing fine elongate hairs, and long flattened setae (Fig. 11E). Abdominal segments IV–VII with sparse, long, appressed pubescence (Fig. 11A).

#### Distribution.

Sichuan (He et al. 2020), Yunnan (Song et al. 2013), Guizhou (Zhang and Hou 2009), Guangxi (Zhou 2001), Guangdong (Wang et al. 2012), Fujian (Zhang and Hou 2009), Hainan (Lee et al. 2020), Taiwan (Seifert 2003).

#### Remarks.

The species *Cardiocondyla wroughtonii* was described as *Emeryia wroughtonii* by Forel (1890) and was later combined in *Cardiocondyla* by Forel (1892). This species has documented records from eight provinces in China; however, because distinguishing these two species *C. obscurior* and *C. wroughtonii* based on the coloration of abdominal segments IV–VII may not be entirely reliable, all literature records outside of Taiwan require further verification.

##### Key to the species of *Cardiocondyla* from China (based on workers)

**Table:** 

1	Antennae 11-segmented	***C. zhoui* sp. nov**.
–	Antennae 12-segmented	**2**
2	Propodeal spines long and thin, in lateral view basal width only half of length (Fig. 12A)	**3**
–	Propodeal spines short and thick, absent or denticulate; in lateral view basal width equal to or less than length (Fig. 12B)	**4**
3	Abdominal segments IV–VII deep black overall, first and second tergites equally colored, contrasting significantly with thorax and head (Fig. 9A)	***C. obscurior***
–	Abdominal segments IV–VII yellow-brown or tawny, not spread evenly, first tergite usually darker than second tergite, contrasting slightly with thorax and head (Fig. 10A)	***C. wroughtonii***
4	Petiolar node high, height in lateral view usually ~2× node length (Fig. 13A)	**5**
–	Petiolar node long, length in lateral view nearly equal to or greater than node height (Fig. 13B)	**10**
5	Clypeus smooth and shining, with only slight ridges restricted to margins. (Fig. 14A)	***C. elegans***
–	Central portion of clypeus with intermittent longitudinal ridges or carinae from epistomal sulcus to anterior margin (Fig. 14B)	**6**
6	With head in full-face view, with strong and sparse longitudinal carinae on malar areas, extending over eyes (Fig. 15A)	***C. gibbosa***
–	With head in full-face view, malar areas with inconspicuous longitudinal carinae, weak or intermixed in reticulated sculptures (Fig. 15B)	**7**
7	With head in full-face view, frontal area smooth and longitudinally striate, not forming a complex reticular sculpture (Fig. 16A). Lateral sides of the pronotum smooth and largely lacking both punctures and carinae	***C. tibetana***
–	With head in full-face view, frontal area rough and densely reticulate. Lateral view of the pronotum bearing at least visible punctures and carinae (Fig. 16B)	**8**
8	In lateral view, mesopleuron and propodeum intersegmental transverse carinae present, but fused with meso-metapleural suture (Fig. 17B)	***C. stambuloffii***
–	In lateral view, mesopleuron and propodeum spanned by several strong, prominently raised intersegmental transverse carinae (Fig. 17A)	**9**
9	Propodeal spines projecting obliquely ventrally in lateral view (Fig. 18A). Anterior junction of petiolar node and peduncle strongly sloping. Anterior clypeal margin straight	***C. koshewnikovi***
–	Propodeal spines projecting obliquely dorsally in lateral view (Fig. 18B). Anterior junction of petiolar node and peduncle more gentle, not obviously steep. Anterior clypeal margin convex	***C. rolandi***
10	Head overall extensively smooth and shining	**11**
–	Head sculptured, sometimes vertex smooth, but frontal and cheeks always rough	**12**
11	In lateral view, pronotum finely reticulate; propodeum exhibits dense and rough sculpture, it is specifically areolate-reticulate across the entire surface, the cells being moderately coarse and contiguous. (Fig. 19A)	***C. nigra***
–	In lateral view, pronotum extensively smooth, with only slight traces of sculpture; propodeum smoothly and weakly sculptured (Fig. 19B)	***C. ulianini***
12	Dorsal view of pronotum extensively smooth and shining (Fig. 20A)	***C. parvinoda***
–	Dorsal view of pronotum strongly and densely reticulate (Fig. 20B)	**13**
13	Lateral view of meso-propodeal sulcus obsolete, only slightly impressed in lateral view (Fig. 21A)	***C. minutior***
–	Lateral view of meso-propodeal sulcus with a clear crease, distinctly impressed in lateral view (Fig. 21B)	**14**
14	Petiolar peduncle short and thick, its length slightly shorter than node (Fig. 22A)	***C. nuda***
–	Petiolar peduncle elongate, its length subequal to node (Fig. 22B)	**15**
15	Anterolateral margin of pronotum in dorsal view with distinct shoulders (Fig. 23 A);	***C. kagutsuchi***
–	Anterolateral margin of pronotum in dorsal view rounded without distinct shoulders (Fig. 23B)	***C. itsukii***

**Figure 12.**
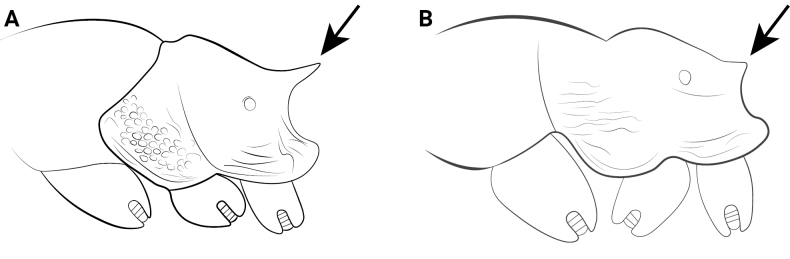
Body of *Cardiocondyla* workers in lateral view. **A**. *C. obscurior*; **B**. *C. elegans*.

**Figure 13.**
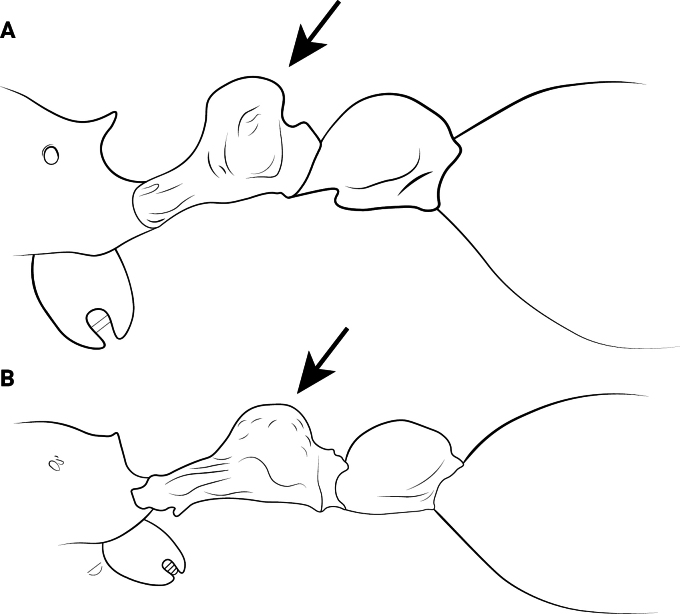
Petiolar node of *Cardiocondyla* workers in lateral view. **A**. *C. elegans*; **B**. *C. nigra*.

**Figure 14.**
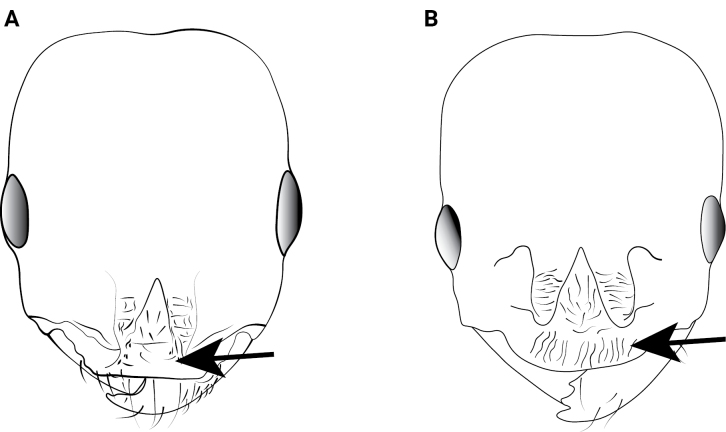
Head of *Cardiocondyla* workers in full-face view, showing the sculpture of the clypeus. **A**. *C. elegans*; **B**. *C. gibbosa*.

**Figure 15.**
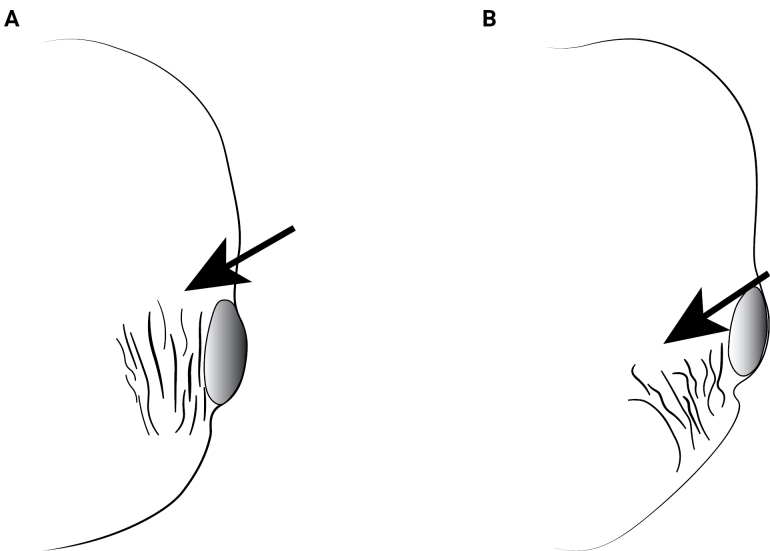
Head of *Cardiocondyla* workers in full-face view, showing the carinae of the on malar areas. **A**. *C. gibbosa*; **B**. *C. tibetana*.

**Figure 16.**
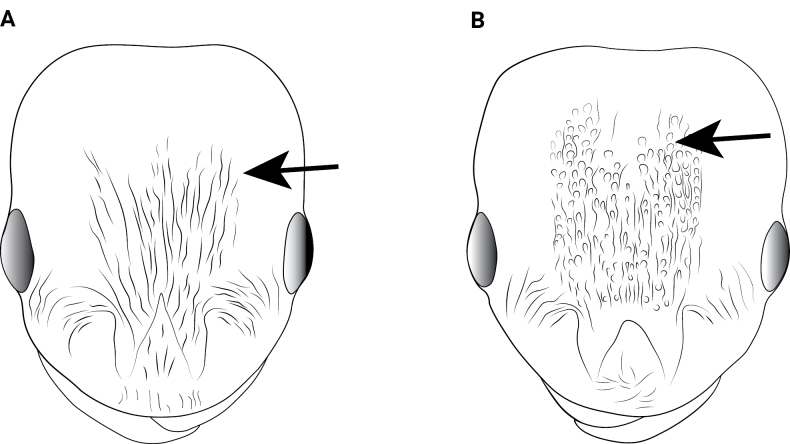
Head of *Cardiocondyla* workers in full-face view, showing the sculpture of frontal area. **A**. *C. tibetana*; **B**. *C. stambuloffii*.

**Figure 17.**
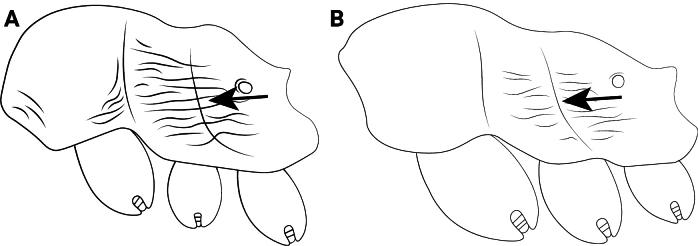
Body of *Cardiocondyla* workers in lateral view, showing the carinae of mesopleuron and propodeum. **A**. *C. koshewnikovi*; **B**. *C. stambuloffii*.

**Figure 18.**
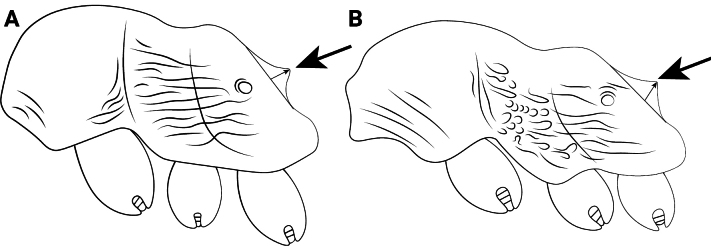
Body of *Cardiocondyla* workers in lateral view, showing the propodeal spines. **A**. *C. koshewnikovi*; **B**. *C. rolandi*.

**Figure 19.**
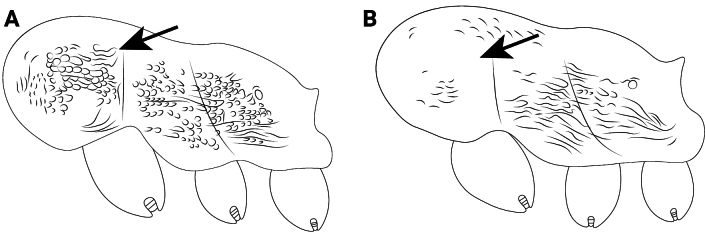
Body of *Cardiocondyla* workers in lateral view. **A**. *C. nigra*; **B**. *C. ulianini*.

**Figure 20.**
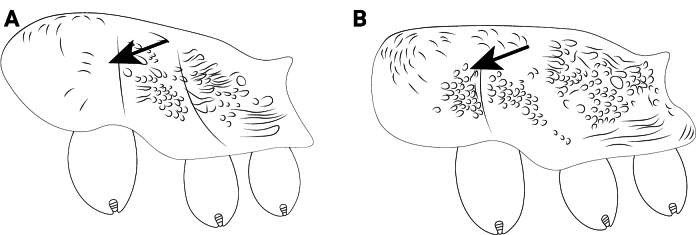
Body of *Cardiocondyla* workers in lateral view. **A**. *C. parvinoda*; **B**. *C. minutior*.

**Figure 21.**
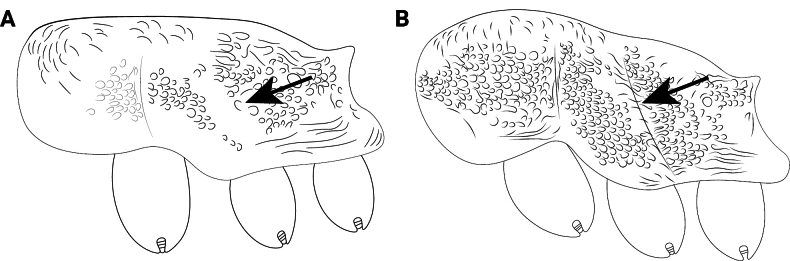
Body of *Cardiocondyla* workers in lateral view. **A**. *C. minutior*; **B**. *C. nuda*.

**Figure 22.**
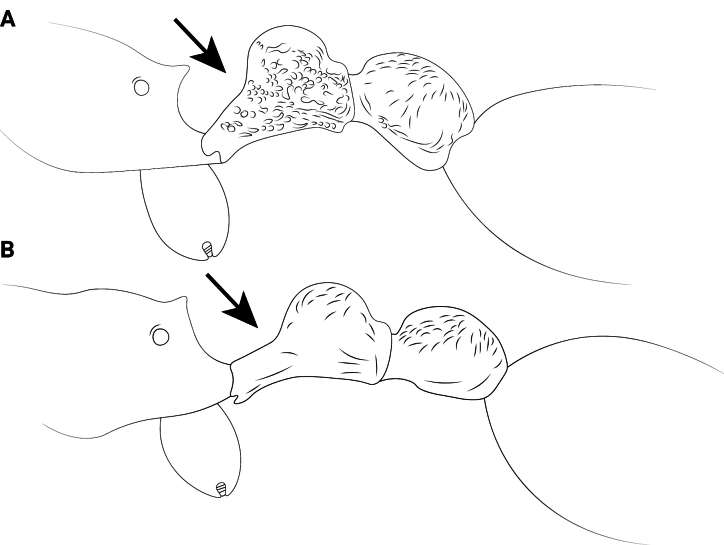
Petiolar node of *Cardiocondyla* workers in lateral view. **A**. *C. nuda*; **B**. *C. itsukii*.

**Figure 23.**
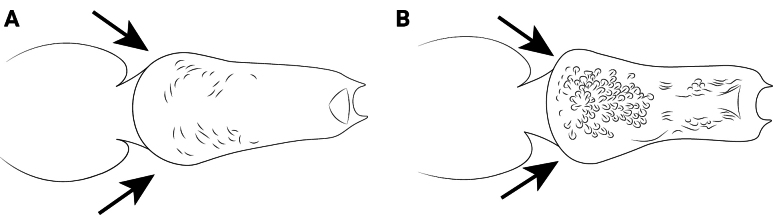
Body of *Cardiocondyla* workers in dorsal view. **A**. *C. kagutsuchi*; **B**. *C. itsukii*.

## Supplementary Material

XML Treatment for Cardiocondyla

XML Treatment for Cardiocondyla nigra

XML Treatment for Cardiocondyla elegans

XML Treatment for Cardiocondyla minutior

XML Treatment for Cardiocondyla parvinoda

XML Treatment for Cardiocondyla itsukii

XML Treatment for Cardiocondyla kagutsuchi

XML Treatment for Cardiocondyla nuda

XML Treatment for Cardiocondyla zhoui

XML Treatment for Cardiocondyla gibbosa

XML Treatment for Cardiocondyla koshewnikovi

XML Treatment for Cardiocondyla rolandi

XML Treatment for Cardiocondyla stambuloffii

XML Treatment for Cardiocondyla tibetana

XML Treatment for Cardiocondyla ulianini

XML Treatment for Cardiocondyla obscurior

XML Treatment for Cardiocondyla wroughtonii

## Additional information

### Use of AI

No use of AI was reported.

### Author ORCIDs

Yi Huang https://orcid.org/0009-0008-5136-0571

Ying Zhong https://orcid.org/0000-0002-6289-4455

Tianqi Wu https://orcid.org/0009-0005-6672-3847

Yuyuan Huang https://orcid.org/0009-0005-3406-4875

Li Tian https://orcid.org/0000-0002-7288-9676

### Data availability

All of the data that support the findings of this study are available in the main text or Supplementary Information.

## Supplementary materials

Supplementary material 1 Measurements of *Cardiocondyla zhoui* sp. nov. Data type xlsx Explanation note Morphometric data for *Cardiocondyla zhoui* sp. nov., based on one holotype and four paratypes. https://binary.pensoft.net/file/1583193This dataset is made available under the Open Database License (http://opendatacommons.org/licenses/odbl/1.0/). The Open Database License (ODbL) is a license agreement intended to allow users to freely share, modify, and use this Dataset while maintaining this same freedom for others, provided that the original source and author(s) are credited. Ying Zhong
